# Supplementary material for: Aridity-driven shift in biodiversity–soil multifunctionality relationships
Source: Nat Commun. 2021 Sep 9;12:5350. doi: 10.1038/s41467-021-25641-0 (PMC8429721; doi:10.1038/s41467-021-25641-0)
Supplement: Supplementary file 1 — Supplementary Information [file 41467_2021_25641_MOESM1_ESM.pdf]

Supplementary Information for

Aridity-driven shift in biodiversity–soil multifunctionality relationships

Weigang Hu[1], Jinzhi Ran[1], Longwei Dong[1], Qiajun Du[1], Mingfei Ji[1], Shuran Yao[1], Yuan Sun[1], Chunmei Gong[2], Qingqing Hou[1], Haiyang Gong[1], Renfei Chen[1], Jingli Lu[1], Shubin Xie[1], Zhiqiang Wang[1], Heng Huang[1], Xiaowei Li[1], Junlan Xiong[1], Rui Xia[1], Maohong Wei[1], Dongmin Zhao[1], Yahui Zhang[1], Jinhui Li[1], Huixia Yang[1], Xiaoting Wang[1], Yan Deng[1], Ying Sun[1], Hailing Li[1], Liang Zhang[1], Qipeng Chu[1], Xinwei Li[1], Muhammad Aqeel[1], Abdul Manan[1], Muhammad Adnan Akram[1], Xianghan Liu[1], Rui Li[1], Fan Li[1], Chen Hou[3], Jianquan Liu[1], Jin-Sheng He[1], Lizhe An[1], Richard D. Bardgett[4]*, Bernhard Schmid[5]*, Jianming Deng[1]*

[1]State Key Laboratory of Grassland Agro-Ecosystem, School of Life Sciences, Lanzhou University, Lanzhou 730000, China.
[2]College of Horticulture, Northwest A&F University, Yangling, China.
[3]Department of Biological Science, Missouri University of Science and Technology, Rolla, MO 65409, USA.
[4]Department of Earth and Environmental Sciences, The University of Manchester, Oxford Road, Manchester M13 9PT, UK.
[5]Department of Geography, Remote Sensing Laboratories, University of Zurich, Winterthurerstrasse 190, CH-8057, Zurich, Switzerland.

These authors contributed equally: Weigang Hu, Jinzhi Ran, Longwei Dong.

*Correspondence to: richard.bardgett@manchester.ac.uk (R.D.B.); bernhard.schmid@ieu.uzh.ch (B.S.); dengjm@lzu.edu.cn (J.D.).

This PDF file includes:
Supplementary Figs. 1–31
Supplementary Tables 1–11
Supplementary References

a

Treatment	Numbers of microcosms	Moisture level (% of field capacity)	Moisture content (%)
1	3	3	2.03 ± 0.034
2	3	5	2.08 ± 0.11
3	3	8	2.75 ± 0.26
4	3	10	3.31 ± 0.33
5	3	20	6.09 ± 0.39
6	3	40	11.58 ± 1.12
7	3	60	17.05 ± 1.21
8	3	80	23.28 ± 1.72
9	3	100	28.38 ± 1.20
10	3	120	33.57 ± 1.94

b

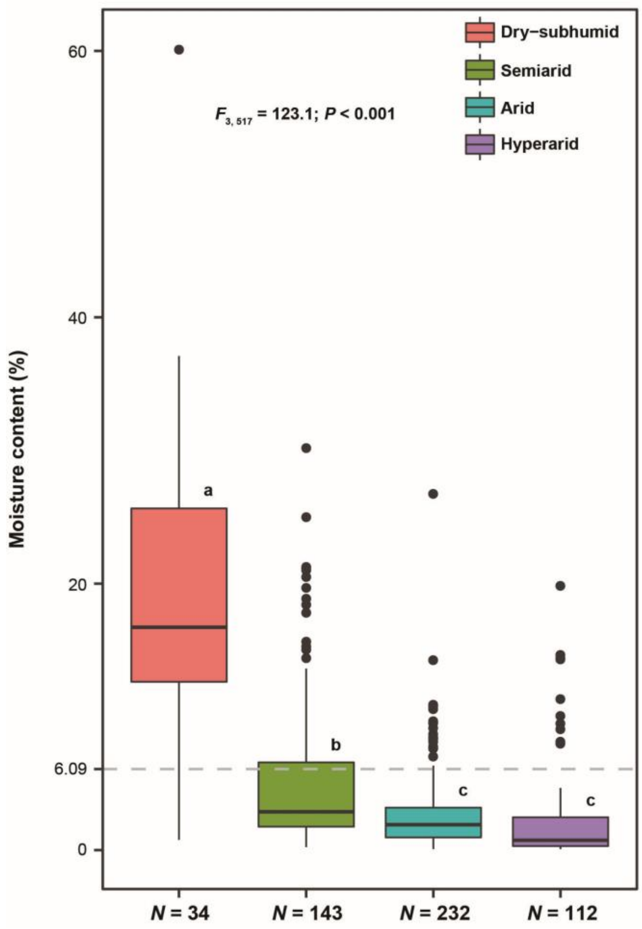

Supplementary Fig. 1. Design of microcosm experiment. a Design of soil microcosm experiment. Thirty experimental microcosms composed of ten moisture levels with three replicates were established. The moisture content (mean ± SE) of different treatments matched well with the differences in moisture conditions among a subset of field soil samples (*N* = 521; **b**). **b** Boxplots demonstrate the differences in moisture content of a subset of field soil samples among the four different dryland subtypes. Boxplots show the median (centre line), 25th and 75th percentiles of each distribution. Whiskers represent the minimum and maximum values that remain inferior 1.5 times the interquartile range below or above the distribution median. Outliers

are indicated by filled black points. Significant differences are determined using one-way ANOVA followed by a Tukey's HSD multiple comparison test (two-sided $P \leq 0.05$) and indicated by lowercase letters. Dashed line denotes the mean value of moisture content (i.e., 6.09%) measured at the 20% field capacity created in the microcosm experiment.

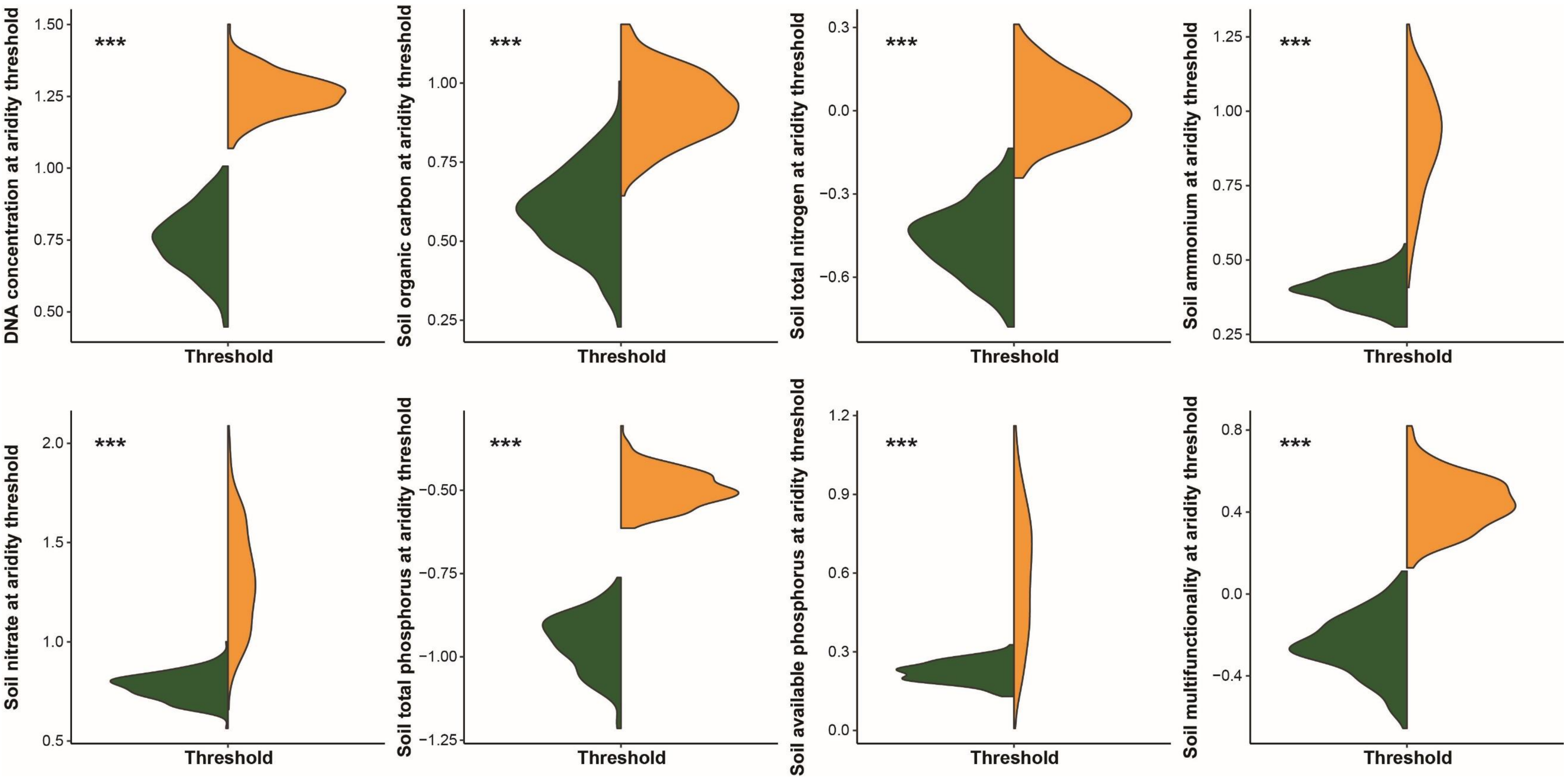

Supplementary Fig. 2. Differences in the predicted values of each of seven individual soil functions and multifunctionality at the aridity threshold. Violin diagrams show bootstrapped predicted values at the threshold of the two regressions existing at each side of the aridity threshold found for each of seven individual soil functions and multifunctionality in Fig. 2b–i of the main text (dark green for the regressions before the threshold and orange for the regressions after the threshold). Significant differences between before and after the threshold are determined using an unpaired two-sided Mann-Whitney U test. Significance level is: ***$P < 0.001$.

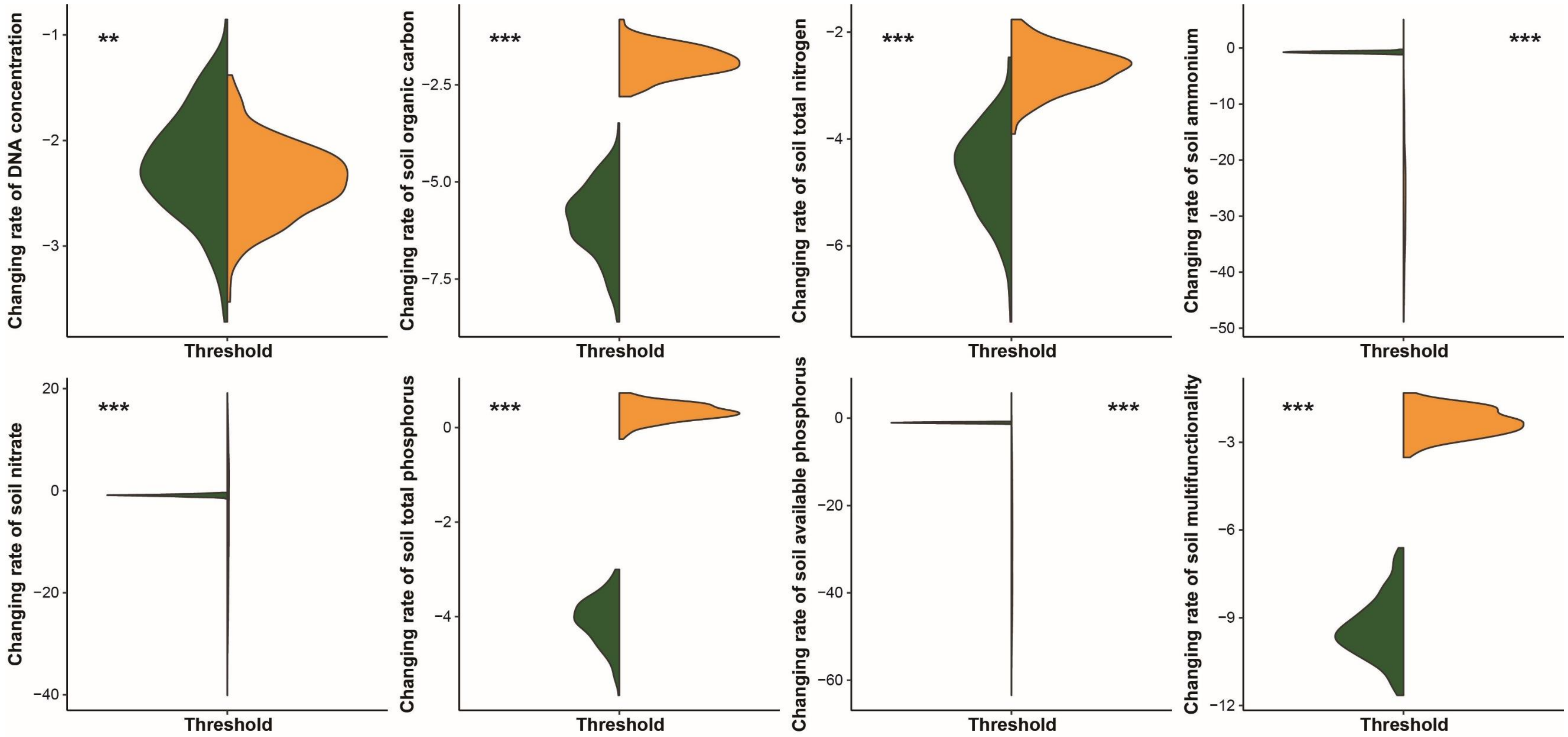

Supplementary Fig. 3. Differences in the changing rates of each of seven individual soil functions and multifunctionality at both sides of the aridity threshold. Violin diagrams show bootstrapped slopes of the two regressions existing at each side of the aridity threshold found for each of seven individual soil functions and multifunctionality in Fig. 2b–i of the main text (dark green for the regressions before the threshold and orange for the regressions after the threshold). Significant differences between before and after the threshold are determined using an unpaired two-sided Mann-Whitney U test. Significance levels are: $^{**}P < 0.01$; $^{***}P < 0.001$.

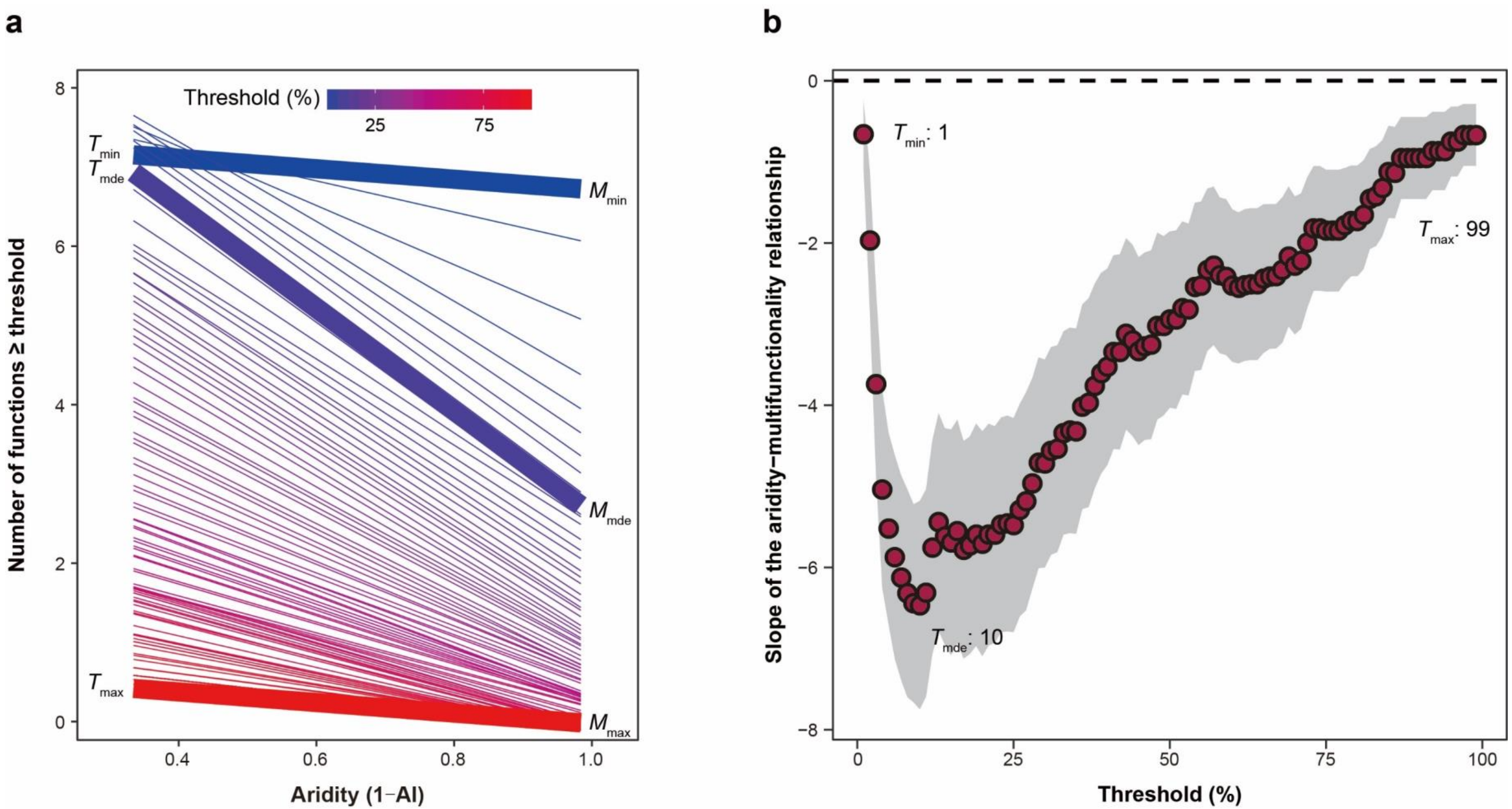

Supplementary Fig. 4. Relationship between aridity and multiple-threshold multifunctionality. Relationships between aridity [1 – aridity index (AI)] and the number of soil functions above a series of sequential thresholds (from 1 to 99% at 1% intervals) of the maximum observed soil function (**a**), and the slopes of these relationships (**b**). T_{min} and T_{max} represent the lowest and highest thresholds whose slopes are significantly different from zero, respectively. T_{mde} is the threshold with the steepest slope. All indices preceded by *M* indicate the number of soil functions (i.e., multiple-threshold multifunctionality) achieving at the corresponding thresholds.

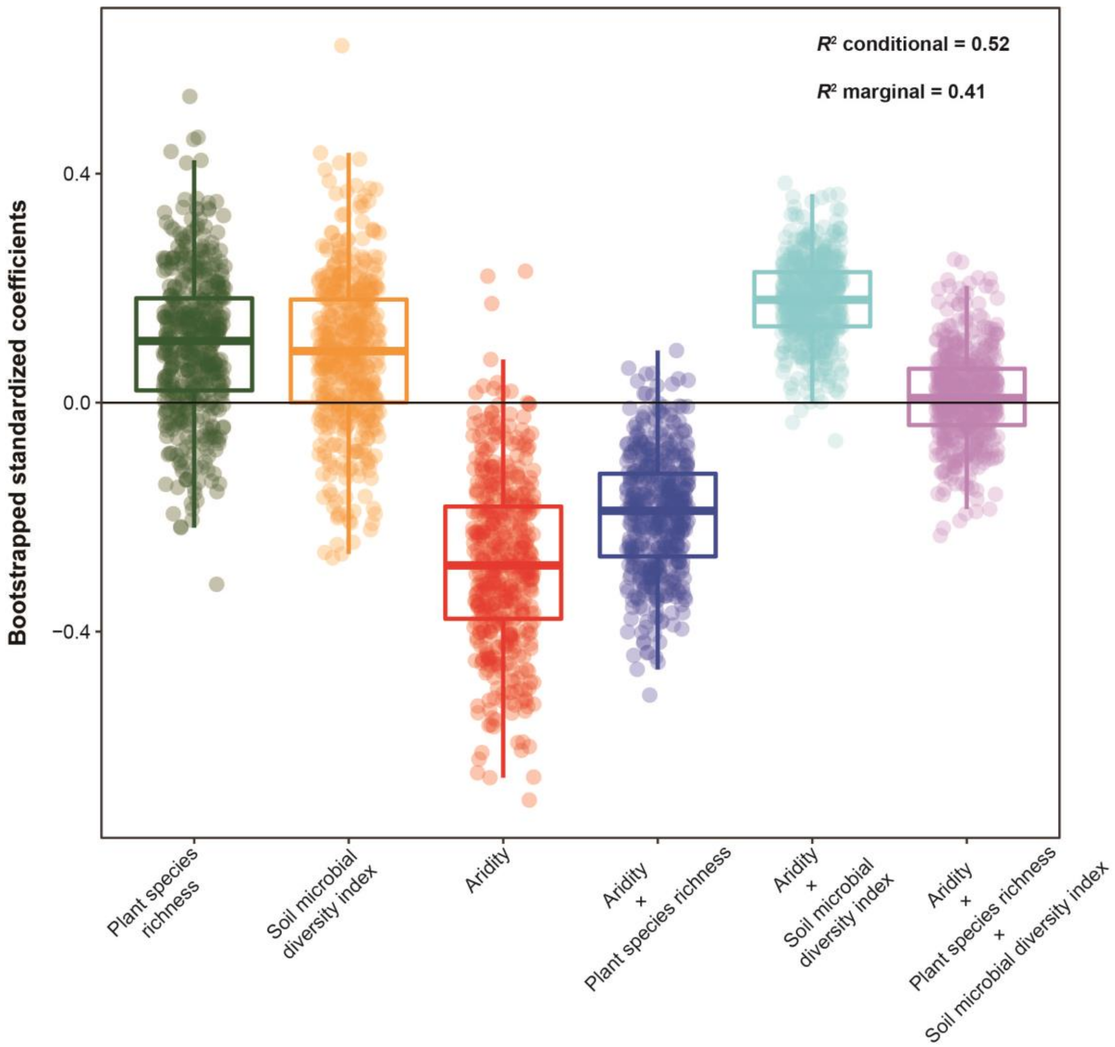

Supplementary Fig. 5. Bootstrapped standardized coefficients of the fixed terms obtained from a linear mixed-effects model (Equation 3 in the main text). Boxplots show the median (centre line), 25th and 75th percentiles of each distribution (N = 500 independent simulations). Whiskers represent the minimum and maximum values that remain inferior 1.5 times the interquartile range below or above the distribution median. Marginal (variance explained by fixed terms) and conditional (variance explained by fixed and random terms) R^2 values are given.

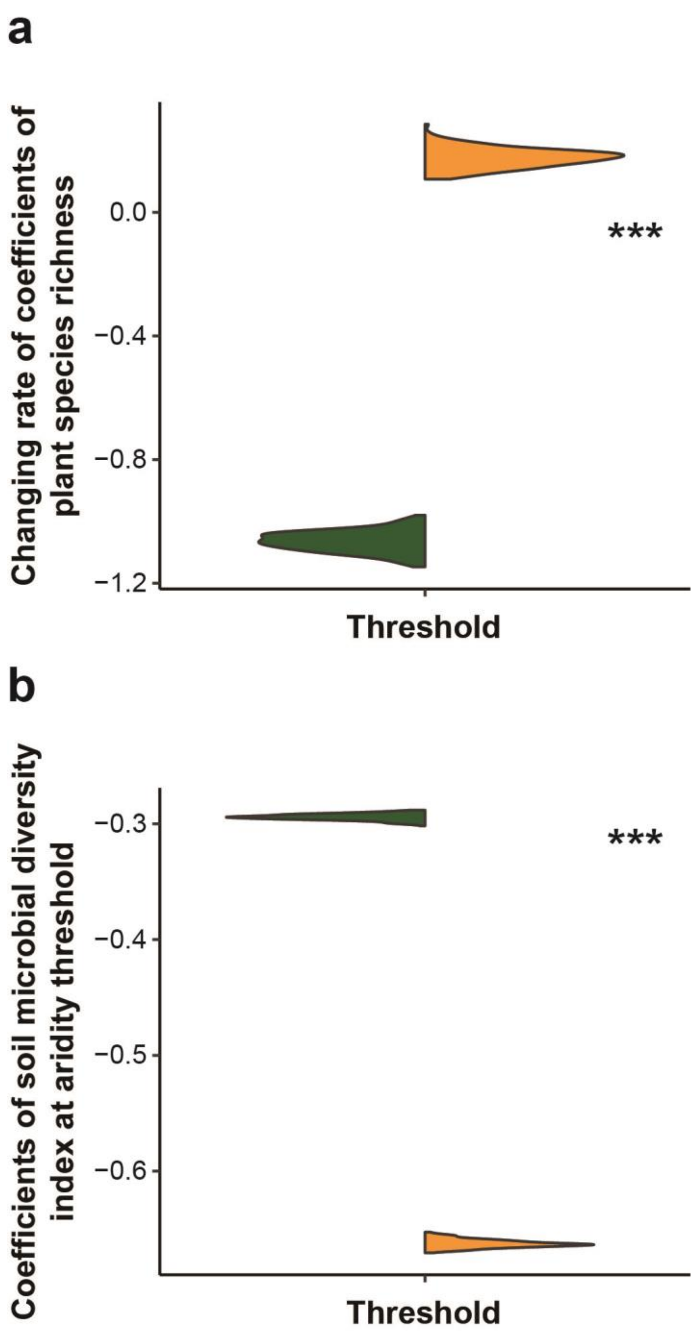

Supplementary Fig. 6. Differences in the changing rates of plant species richness and the predicted values of the soil microbial diversity index at both sides of the aridity threshold. Violin diagrams show bootstrapped slopes (**a**) and bootstrapped predicted values at the threshold (**b**) of the two regressions existing at each side of the aridity threshold found for plant species richness and the soil microbial diversity index in Fig. 3a of the main text, respectively (dark green for the regression before the threshold and orange for the regression after the threshold). Significant differences between before and after the threshold are determined using an unpaired two-sided Mann-Whitney U test. Significance level is: ***$P < 0.001$.

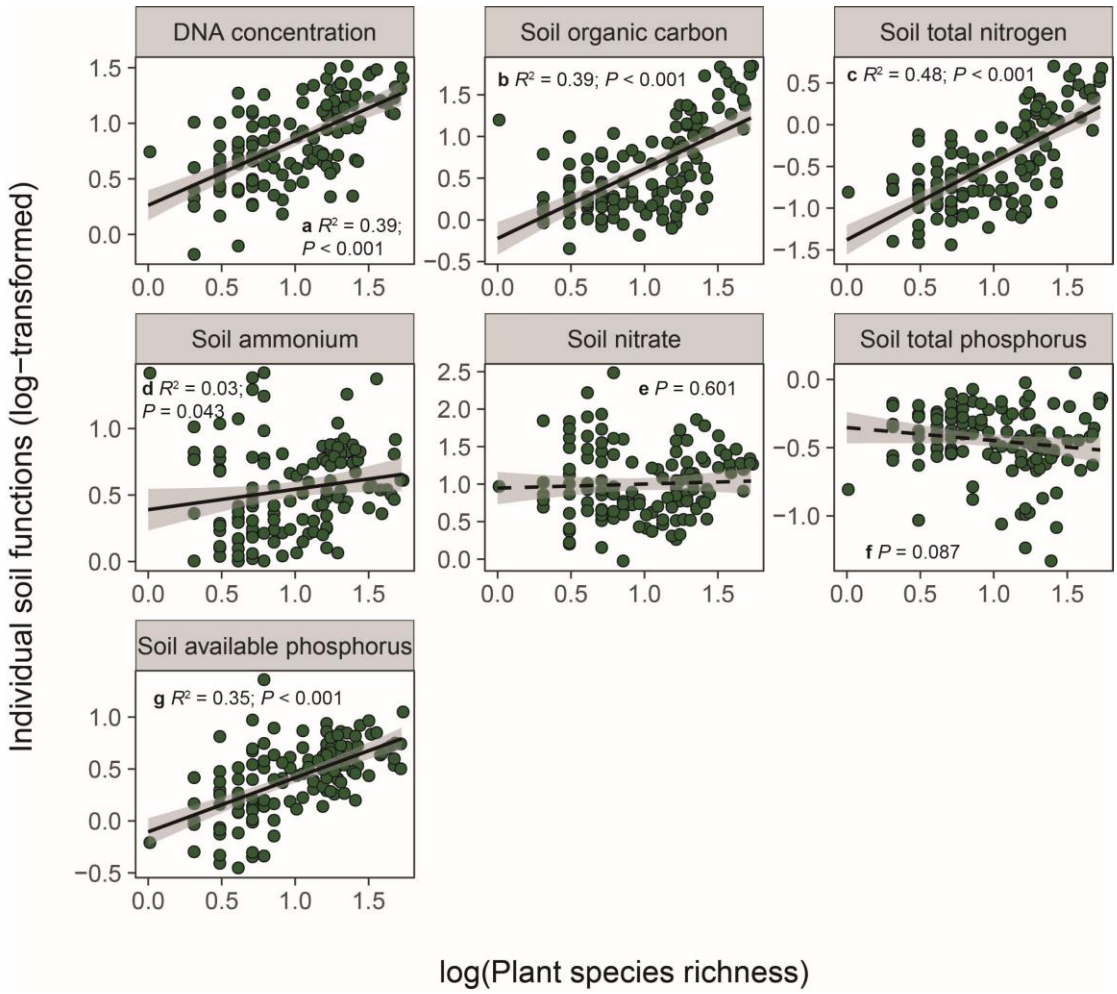

Supplementary Fig. 7. Relationships between log-transformed plant species richness and each of seven individual soil functions. a–g Lines represent the fitted linear ordinary least-squares (OLS) model. Solid and dashed lines denote statistically significant (two-sided $P \leq 0.05$) and non-significant (two-sided $P > 0.05$) relationships, respectively. Shaded areas denote the 95% confidence interval of the regression lines.

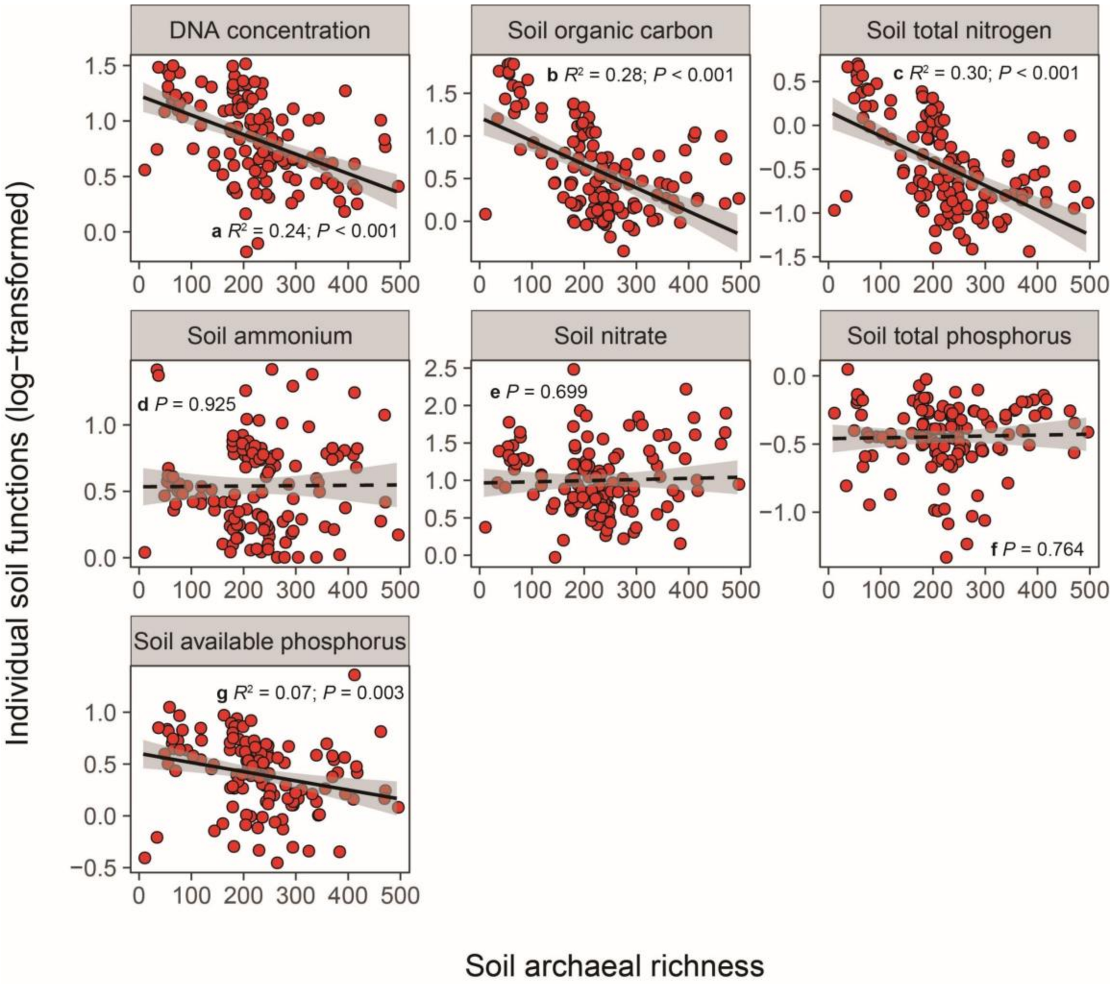

Supplementary Fig. 8. Relationships between soil archaeal richness and each of seven individual soil functions. a–g Lines represent the fitted linear OLS model. Solid and dashed lines denote statistically significant (two-sided $P \leq 0.05$) and non-significant (two-sided $P > 0.05$) relationships, respectively. Shaded areas denote the 95% confidence interval of the regression lines.

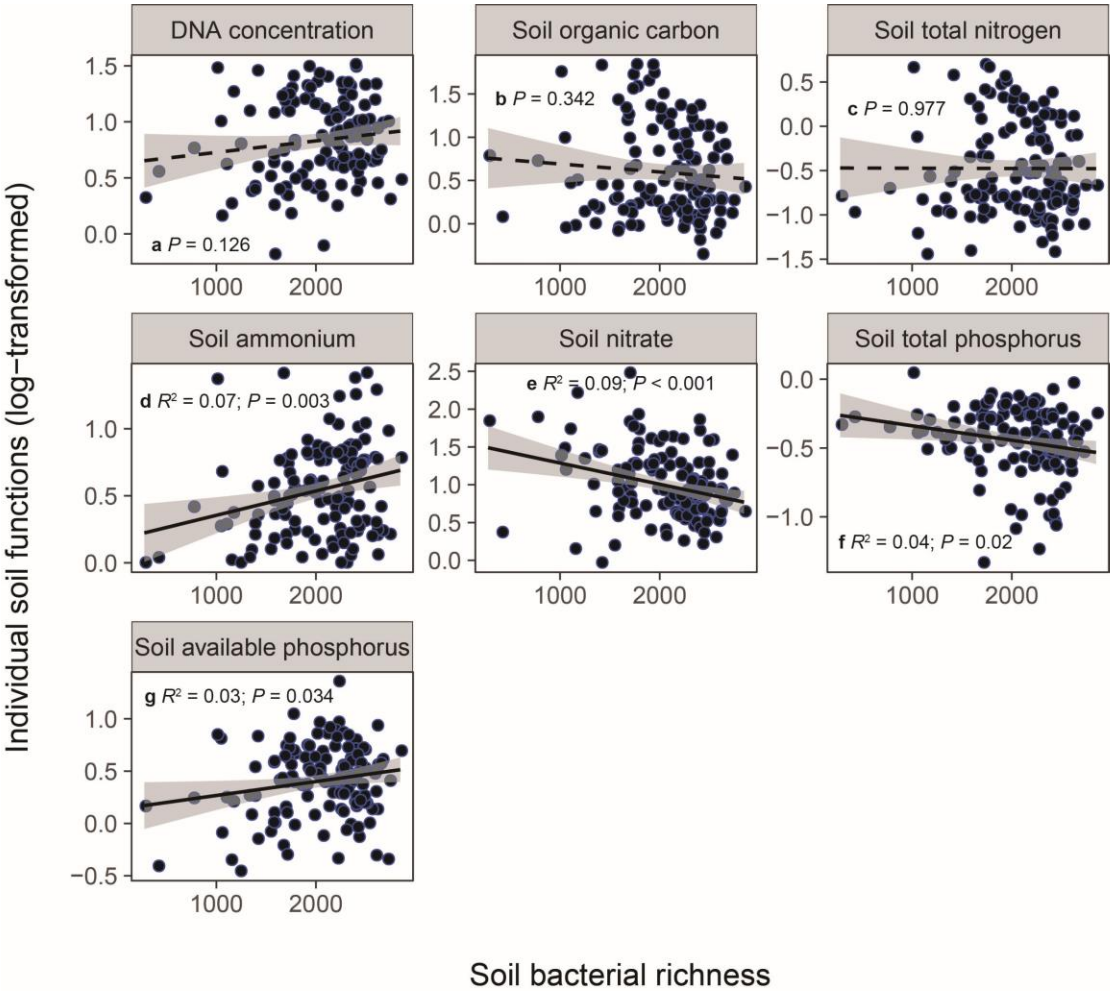

Supplementary Fig. 9. Relationships between soil bacterial richness and each of seven individual soil functions. a–g Lines represent the fitted linear OLS model. Solid and dashed lines denote statistically significant (two-sided $P \leq 0.05$) and non-significant (two-sided $P > 0.05$) relationships, respectively. Shaded areas denote the 95% confidence interval of the regression lines.

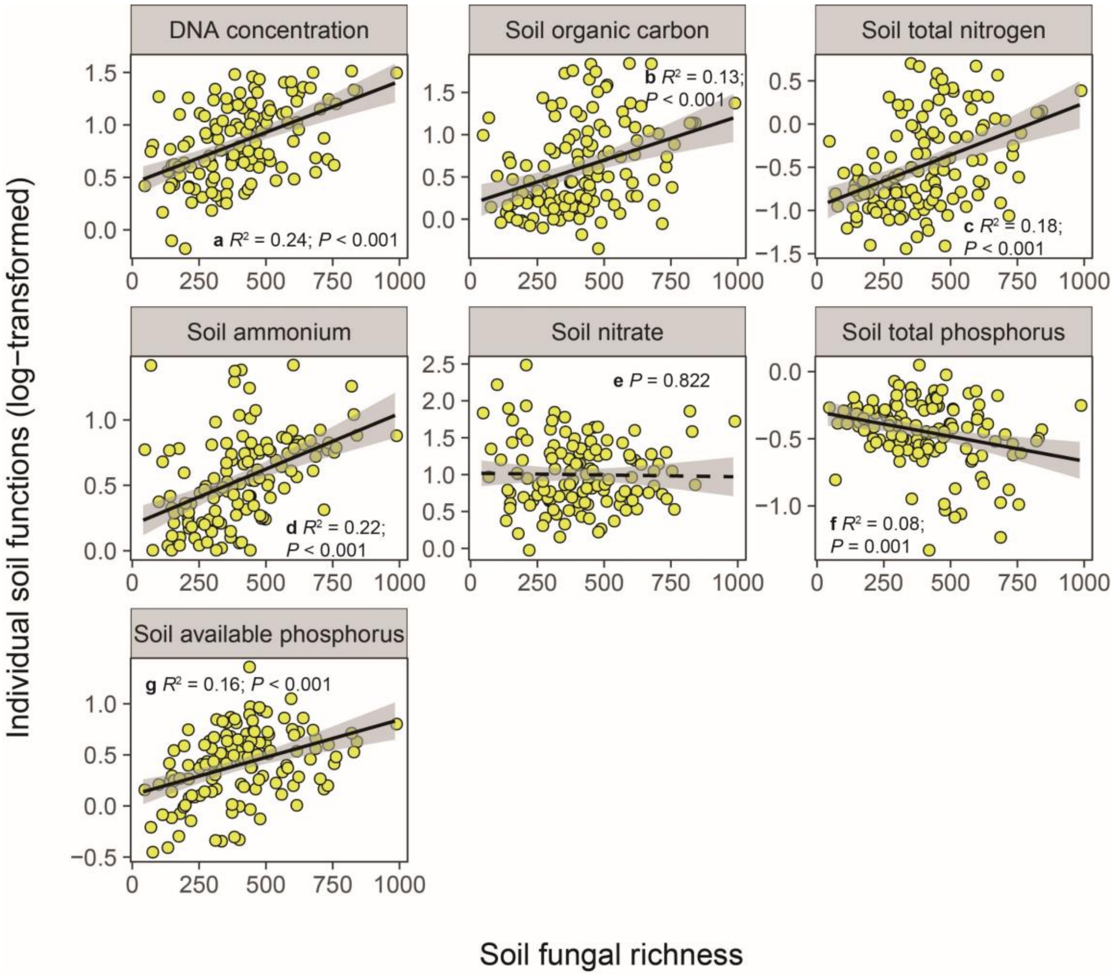

Supplementary Fig. 10. Relationships between soil fungal richness and each of seven individual soil functions. a–g Lines represent the fitted linear OLS model. Solid and dashed lines denote statistically significant (two-sided $P \leq 0.05$) and non-significant (two-sided $P > 0.05$) relationships, respectively. Shaded areas denote the 95% confidence interval of the regression lines.

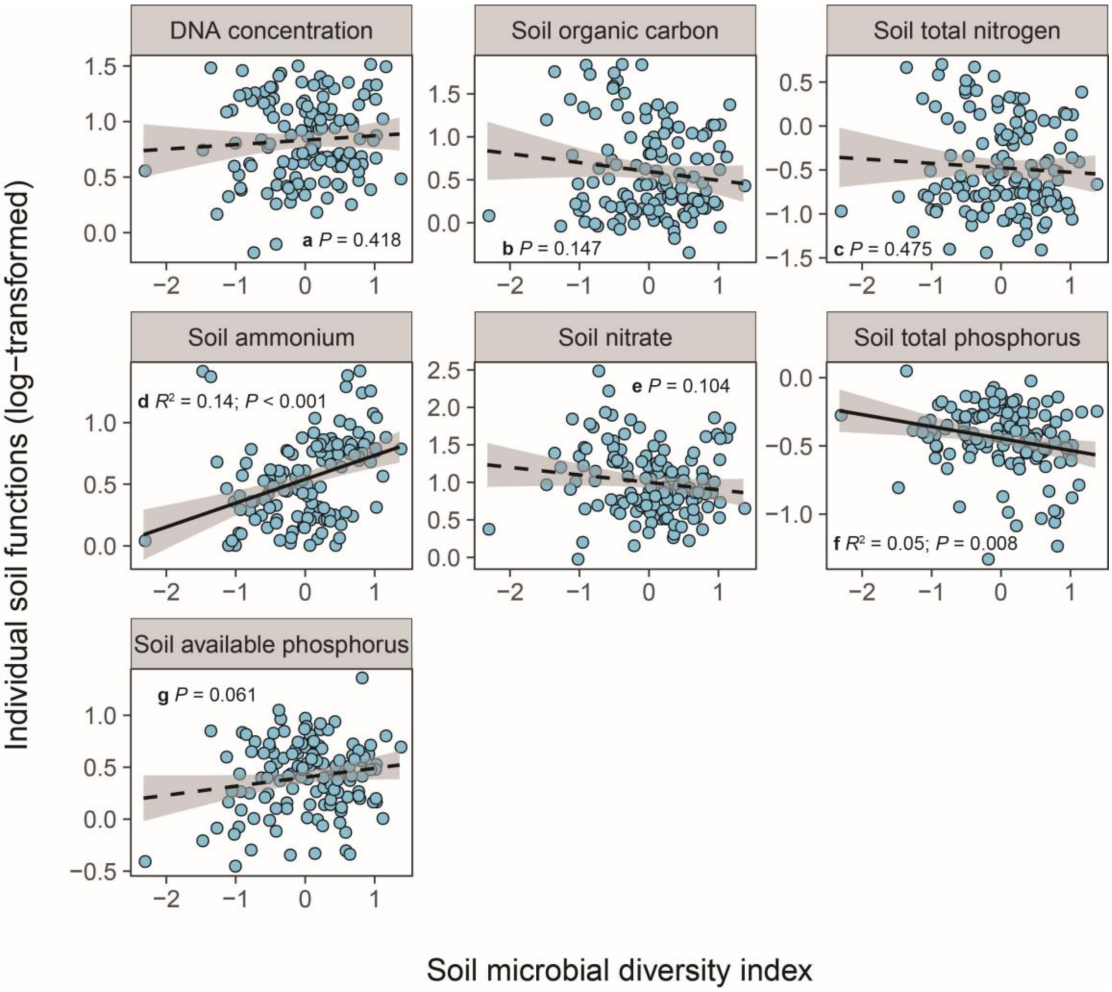

Supplementary Fig. 11. Relationships between the soil microbial diversity index and each of seven individual soil functions. a–g Lines represent the fitted linear OLS model. Solid and dashed lines denote statistically significant (two-sided $P \leq 0.05$) and non-significant (two-sided $P >$ 0.05) relationships, respectively. Shaded areas denote the 95% confidence interval of the regression lines.

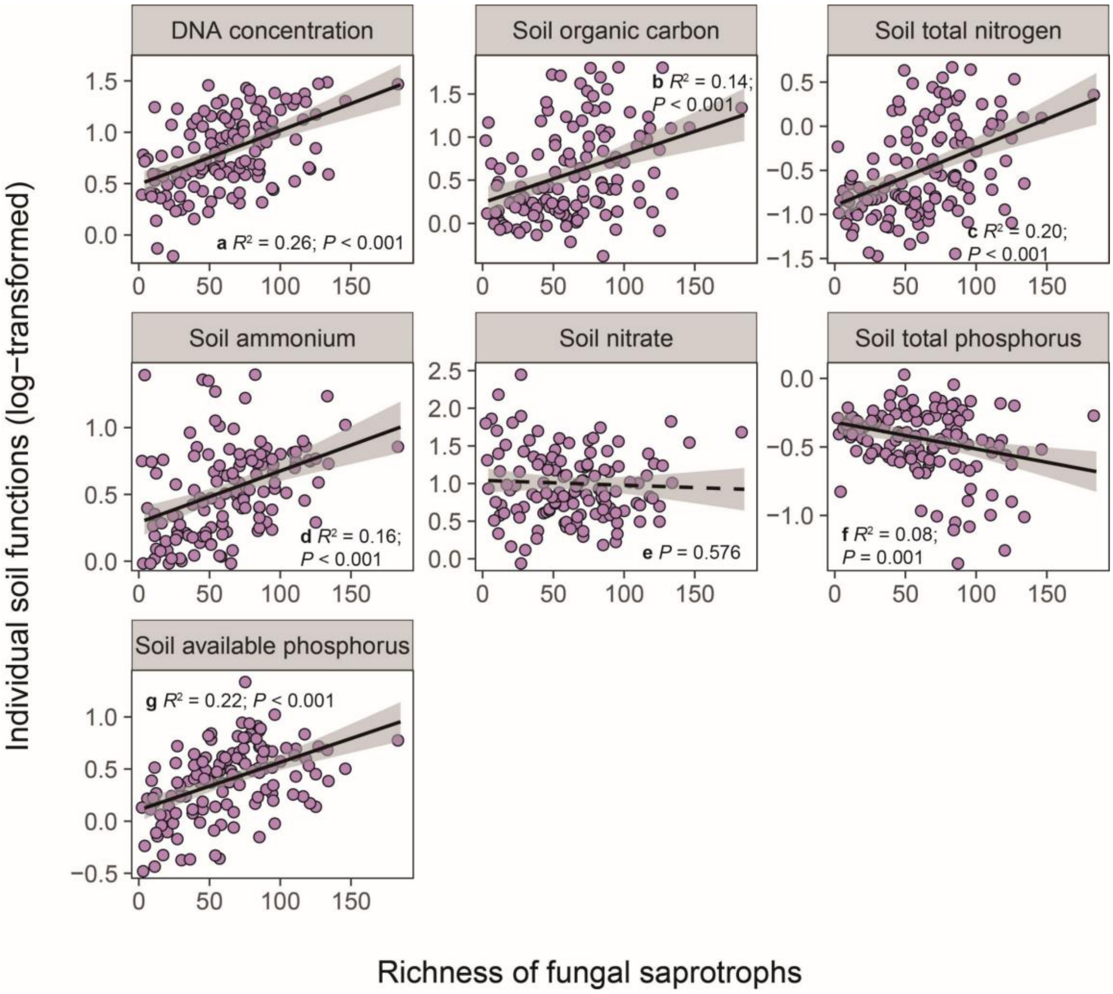

Supplementary Fig. 12. Relationships between richness of fungal saprotrophs and each of seven individual soil functions. a–g Lines represent the fitted linear OLS model. Solid and dashed lines denote statistically significant (two-sided $P \leq 0.05$) and non-significant (two-sided $P > 0.05$) relationships, respectively. Shaded areas denote the 95% confidence interval of the regression lines.

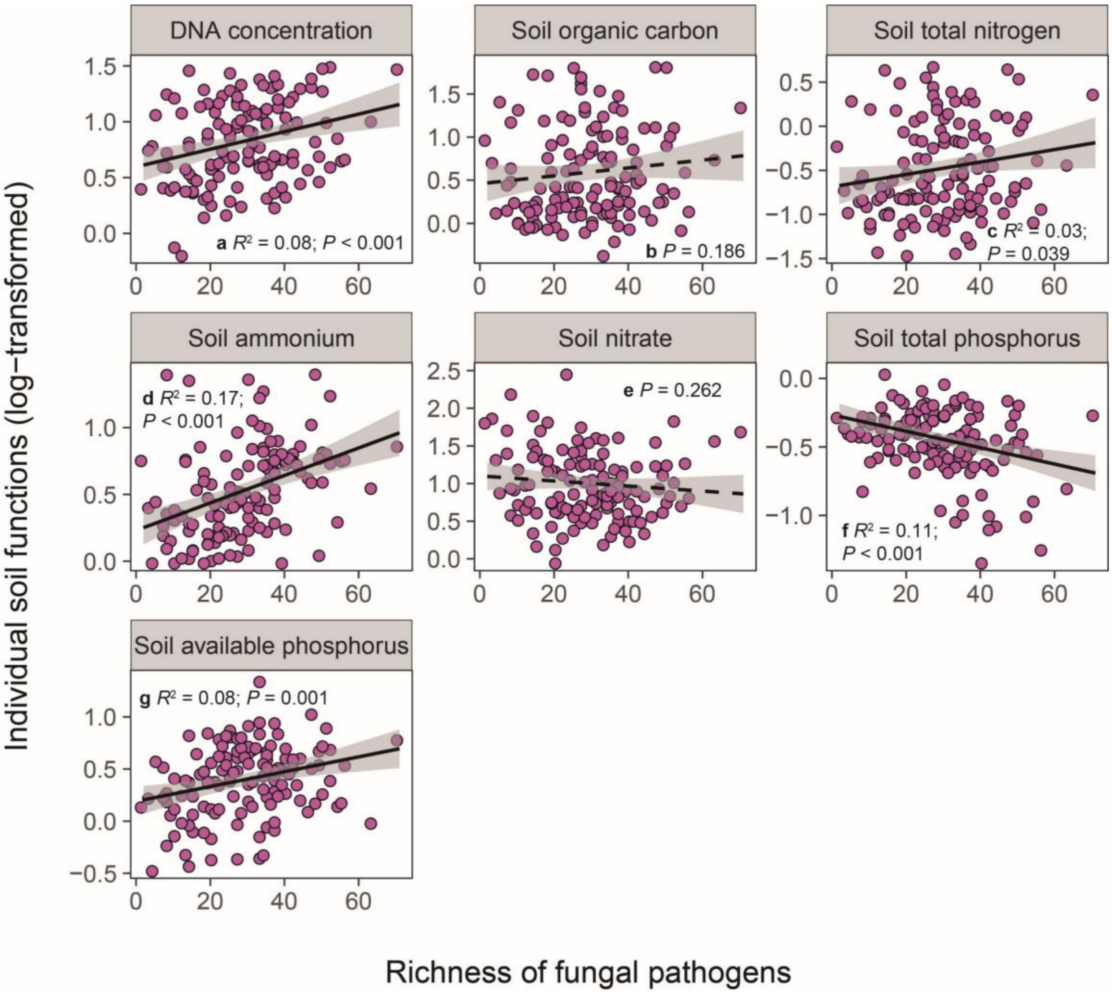

Supplementary Fig. 13. Relationships between richness of fungal pathogens and each of seven individual soil functions. a–g Lines represent the fitted linear OLS model. Solid and dashed lines denote statistically significant (two-sided $P \leq 0.05$) and non-significant (two-sided $P > 0.05$) relationships, respectively. Shaded areas denote the 95% confidence interval of the regression lines.

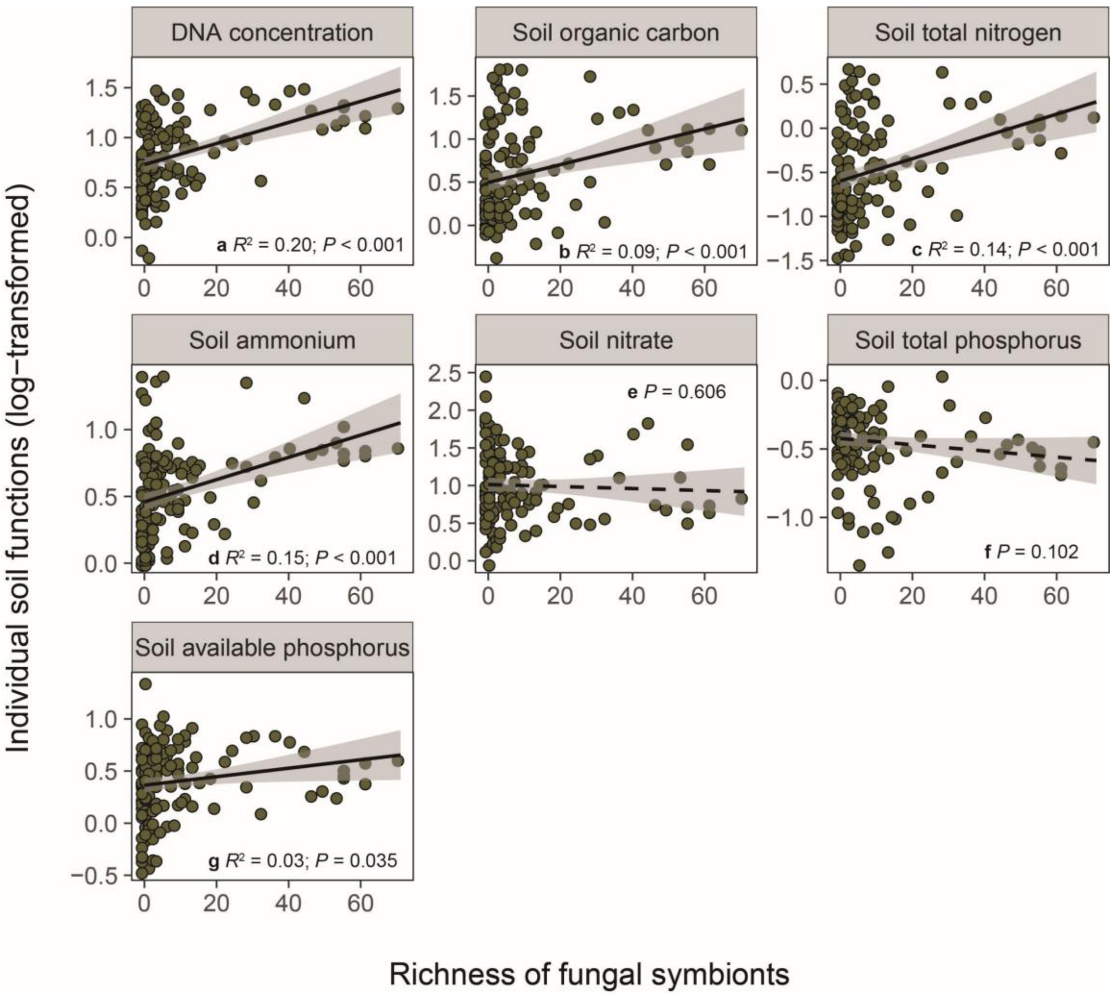

Supplementary Fig. 14. Relationships between richness of fungal symbionts and each of seven individual soil functions. a–g Lines represent the fitted linear OLS model. Solid and dashed lines denote statistically significant (two-sided $P \leq 0.05$) and non-significant (two-sided $P > 0.05$) relationships, respectively. Shaded areas denote the 95% confidence interval of the regression lines.

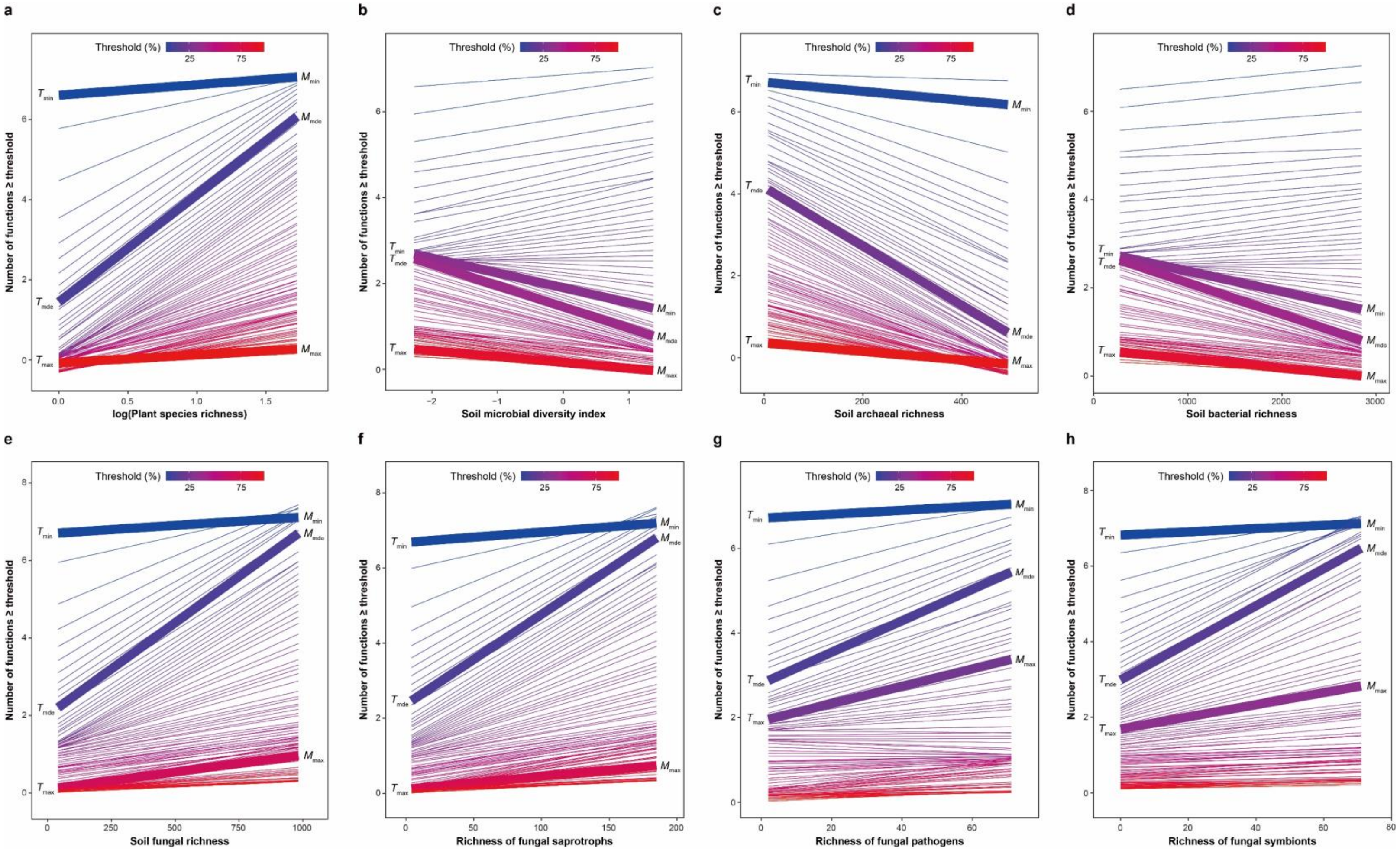

Supplementary Fig. 15. Relationships between biodiversity and multiple-threshold multifunctionality. a–h Relationships between log-transformed plant species richness (**a**), the soil microbial diversity index (**b**), soil archaeal richness (**c**), soil bacterial richness (**d**), soil fungal richness (**e**), richness of fungal saprotrophs (**f**), pathogens (**g**), and symbionts (**h**) and the number of soil functions above multiple thresholds of the maximum observed soil function. Rest of legend as in Supplementary Fig. 4.

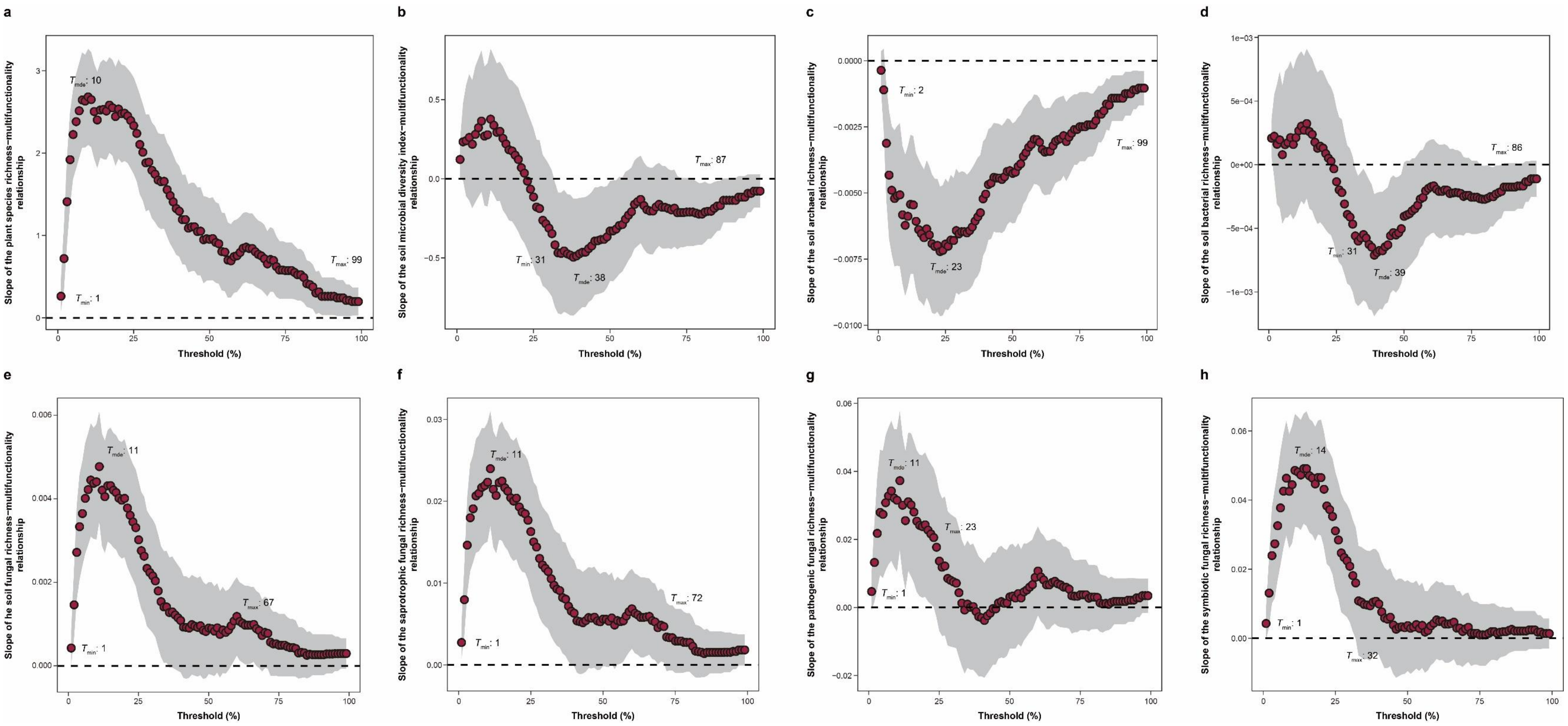

Supplementary Fig. 16. Slopes of the relationships between biodiversity and multiple-threshold multifunctionality. a–h Slopes of the relationships between log-transformed plant species richness (**a**), the soil microbial diversity index (**b**), soil archaeal richness (**c**), soil bacterial richness (**d**), soil fungal richness (**e**), richness of fungal saprotrophs (**f**), pathogens (**g**), and symbionts (**h**) and the number of soil functions above the continuous thresholds from 1 to 99% of the maximum observed soil function. $T_{\min}$, T_{mde} and $T_{\max}$ correspond to those key thresholds depicted in Supplementary Fig. 15. Rest legends as in Supplementary Fig. 4.

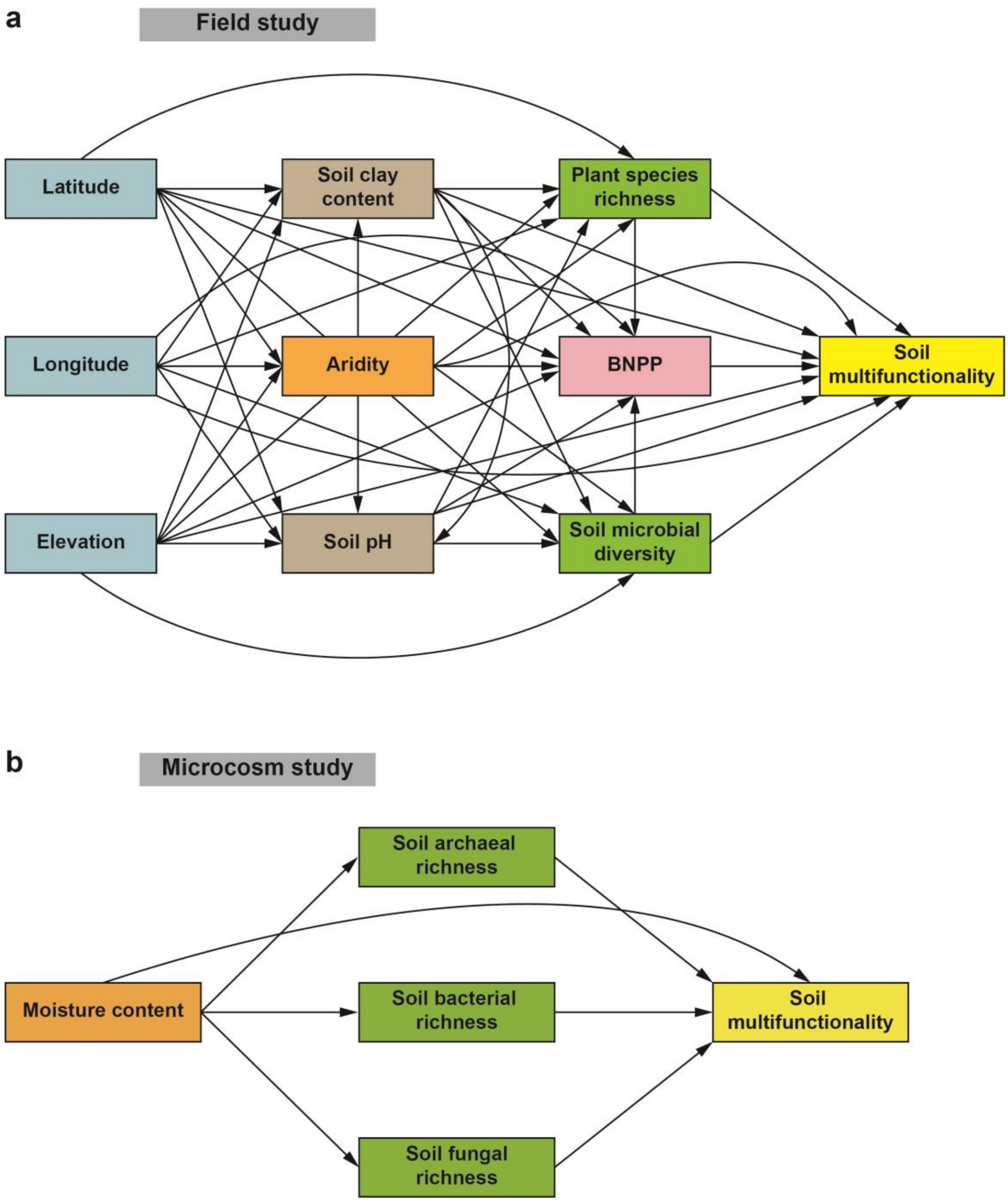

Supplementary Fig. 17. *A priori* structural equation models (SEMs) developed in this study. a An *a priori* SEM developed for the field study. Latitude, longitude, and elevation of the field sites are included to account for the spatial structure of our dataset. BNPP, belowground net primary productivity. **b** An *a priori* SEM developed for the microcosm experiment.

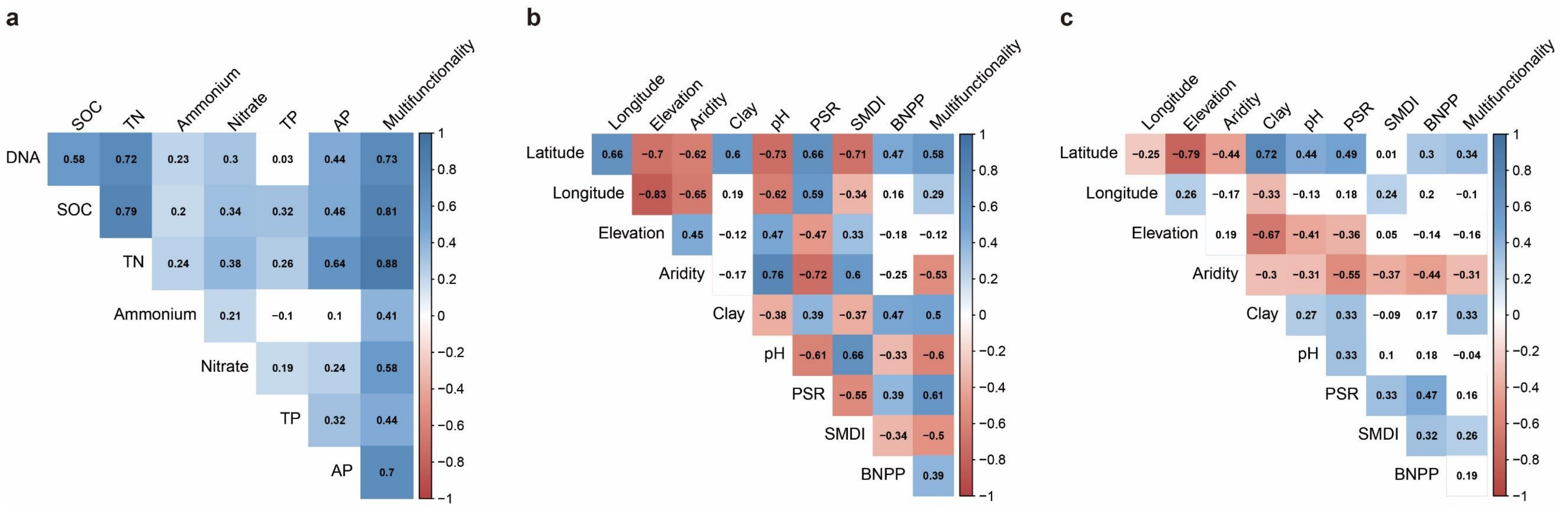

Supplementary Fig. 18. Pearson's correlation matrix for individual soil functions and multifunctionality and for geography, aridity, soil properties, biodiversity, BNPP and soil multifunctionality. a Pearson's correlation matrix for the seven individual soil functions and multifunctionality accounts for potential trade-offs and redundancy among soil functions. DNA, DNA concentration; SOC, soil organic carbon; TN, total soil nitrogen; TP, total soil phosphorus; AP, soil available phosphorus. **b,c** Pearson's correlation matrix for geography, aridity, soil properties, biodiversity, BNPP and soil multifunctionality at sites with aridity < 0.8 (**b**; $N = 54$) and > 0.8 (**c**; $N = 76$), respectively. Clay, soil clay content; PSR, plant species richness; SMDI, soil microbial diversity index. **a–c** Squares present significant positive (blue) or negative (red) and non-significant (blank) values of the corresponding correlation coefficients as shown in the scale bar. Significance levels are determined at $P \leq 0.05$ (two-sided).

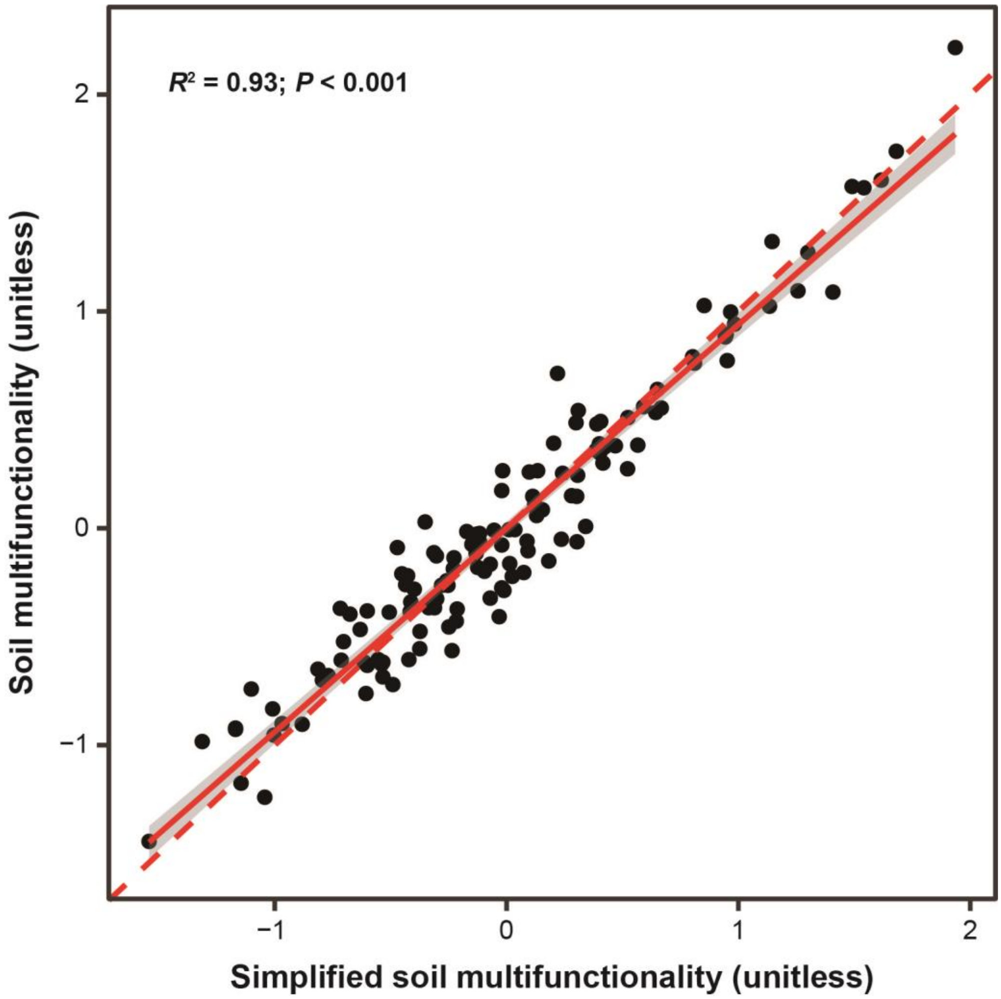

Supplementary Fig. 19. Relationship between soil multifunctionality and simplified soil multifunctionality. Relationship between the soil multifunctionality index calculated with seven soil functions and a simplified version of this index (i.e., simplified soil multifunctionality) without including total soil nitrogen and phosphorus. The solid line represents the fitted linear OLS model and the dashed line indicates the 1:1 line. The significance level is determined at $P \leq 0.05$ (two-sided). The shaded area denotes the 95% confidence interval of the regression line.

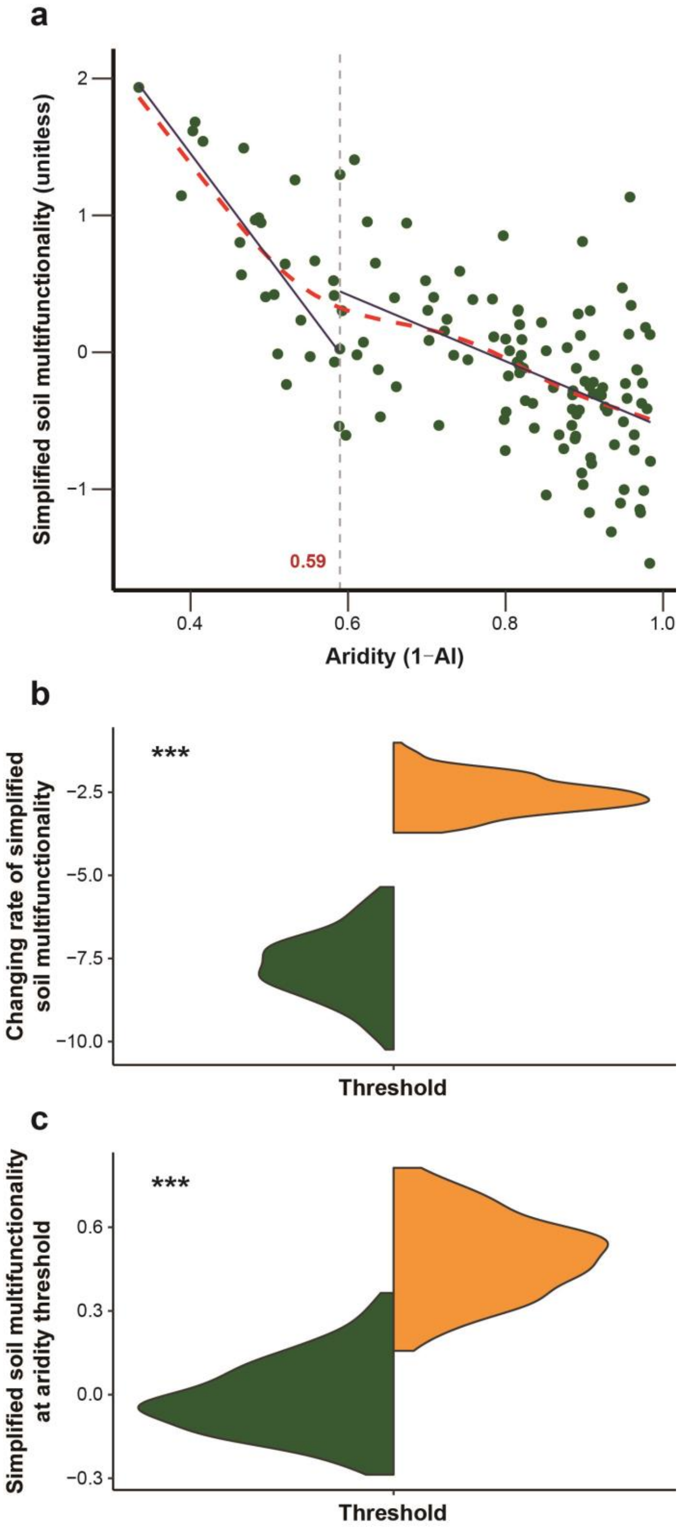

Supplementary Fig. 20. Nonlinear response of simplified soil multifunctionality to aridity. a Nonlinear response of simplified soil multifunctionality to aridity, and its aridity threshold. The red dashed line indicates the nonlinear trend fitted by generalized additive model (GAM). The grey dashed line and inset number in red represent the aridity threshold identified. The blue solid lines denote the linear fits at both sides of the aridity threshold. **b,c** Violin diagrams show bootstrapped slopes (**b**) and bootstrapped predicted values at the threshold (**c**) of the two regressions existing at each side of the aridity threshold found for simplified soil multifunctionality in (**a**). Significant differences between before and after the threshold are determined using an unpaired two-sided Mann-Whitney U test. Rest of legend as in Supplementary Fig. 2.

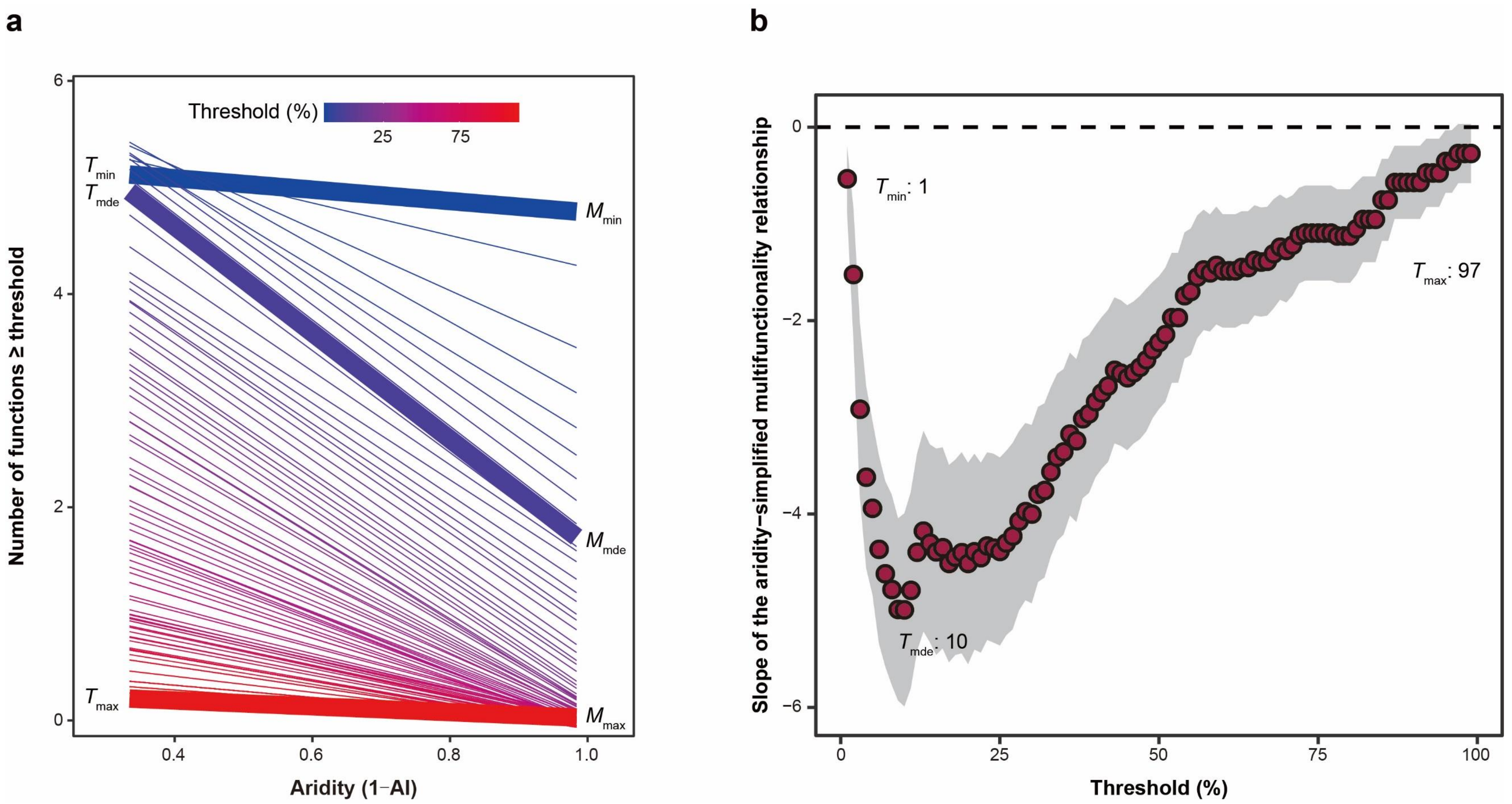

Supplementary Fig. 21. Relationship between aridity and simplified multiple-threshold multifunctionality. Rest of legend as in Supplementary Fig .4.

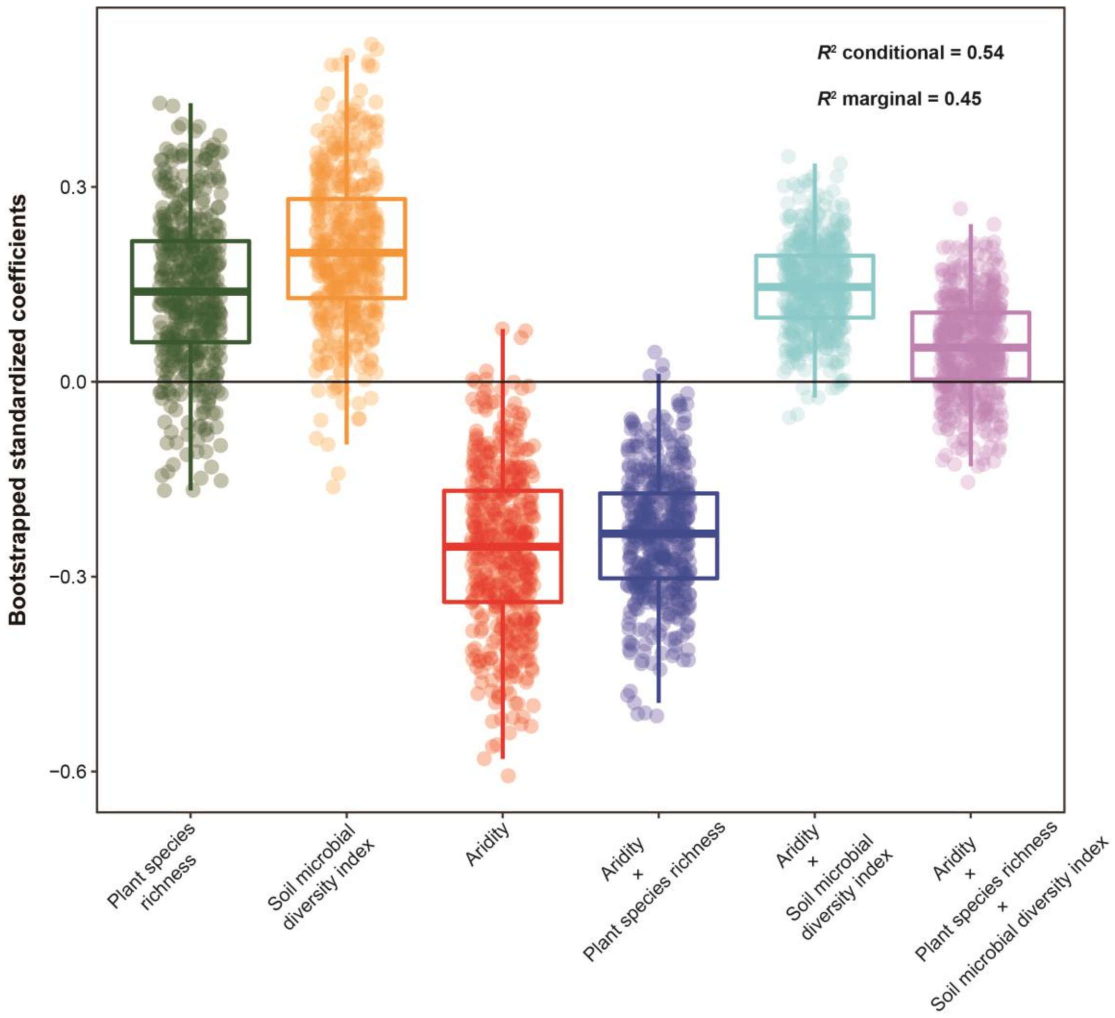

Supplementary Fig. 22. Bootstrapped standardized coefficients of the fixed terms obtained from a linear mixed-effects model (Equation 3 in the main text) applied to simplified soil multifunctionality. Boxplots show the median (centre line), 25th and 75th percentiles of each distribution (N = 500 independent simulations). Whiskers represent the minimum and maximum values that remain inferior 1.5 times the interquartile range below or above the distribution median. Marginal (variance explained by fixed terms) and conditional (variance explained by fixed and random terms) R^2 values are given.

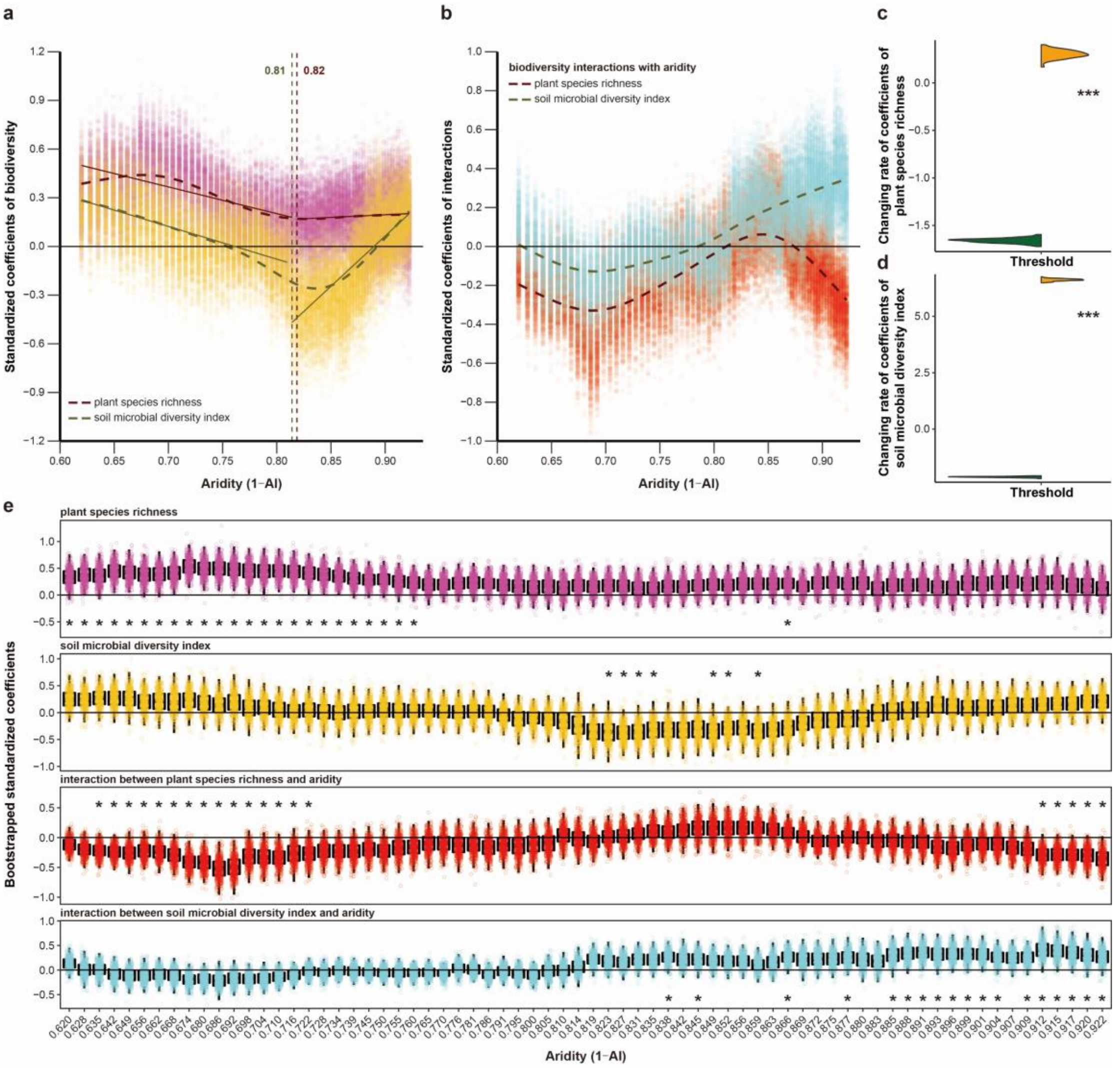

Supplementary Fig. 23. Nonlinear changes of relationships between biodiversity and its interactions with aridity and simplified soil multifunctionality along aridity gradients. a,b Nonlinear changes of standardized coefficients of biodiversity (**a**) and the interactions between biodiversity and aridity (**b**) obtained from a linear mixed-effects model (Equation 3 in the main text) applied to simplified soil multifunctionality throughout a moving subset window of the field sites surveyed along aridity gradients. The dots indicate the bootstrapped coefficients of the fixed terms shown for each subset window. The dashed lines denote the nonlinear trend fitted by GAMs. In (**a**), the vertical dashed lines and inset numbers represent the aridity thresholds identified, and the solid lines represent the linear fits at both sides of each aridity threshold. **c,d** Violin diagrams show bootstrapped slopes of the two regressions existing at each side of the aridity threshold found for plant species richness and the soil microbial diversity index in (**a**), respectively. Significant differences between before and after the threshold are determined using an unpaired two-sided Mann-Whitney U test. Rest of legend as in Supplementary Fig. 2. **e** Boxplots demonstrate the distribution of bootstrapped standardized coefficients corresponding to those in (**a,b**) for each subset window (N = 500 independent simulations). Boxplots show the median (centre line), 25th and 75th percentiles of each distribution. Whiskers represent the minimum and maximum values that remain inferior 1.5 times the interquartile range below or above the distribution median. Asterisks indicate significant values of coefficients at 95% confidence intervals (one-sided $P \leq 0.05$).

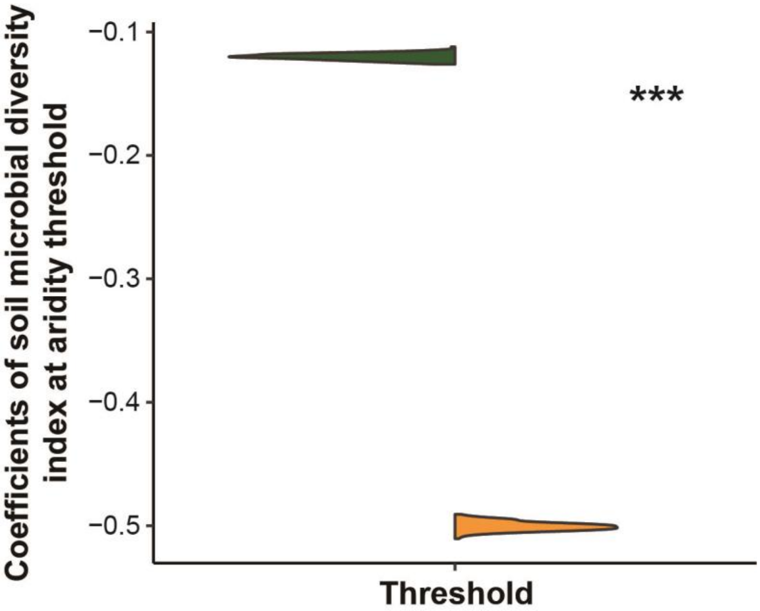

Supplementary Fig. 24. Differences in the predicted values of the soil microbial diversity index at both sides of the aridity threshold. Violin diagrams show bootstrapped predicted values at the threshold of the two regressions existing at each side of the aridity threshold found for the soil microbial diversity index in Supplementary Fig. 23a. Significant differences between before and after the threshold are determined using an unpaired two-sided Mann-Whitney U test. Rest of legend as in Supplementary Fig. 2.

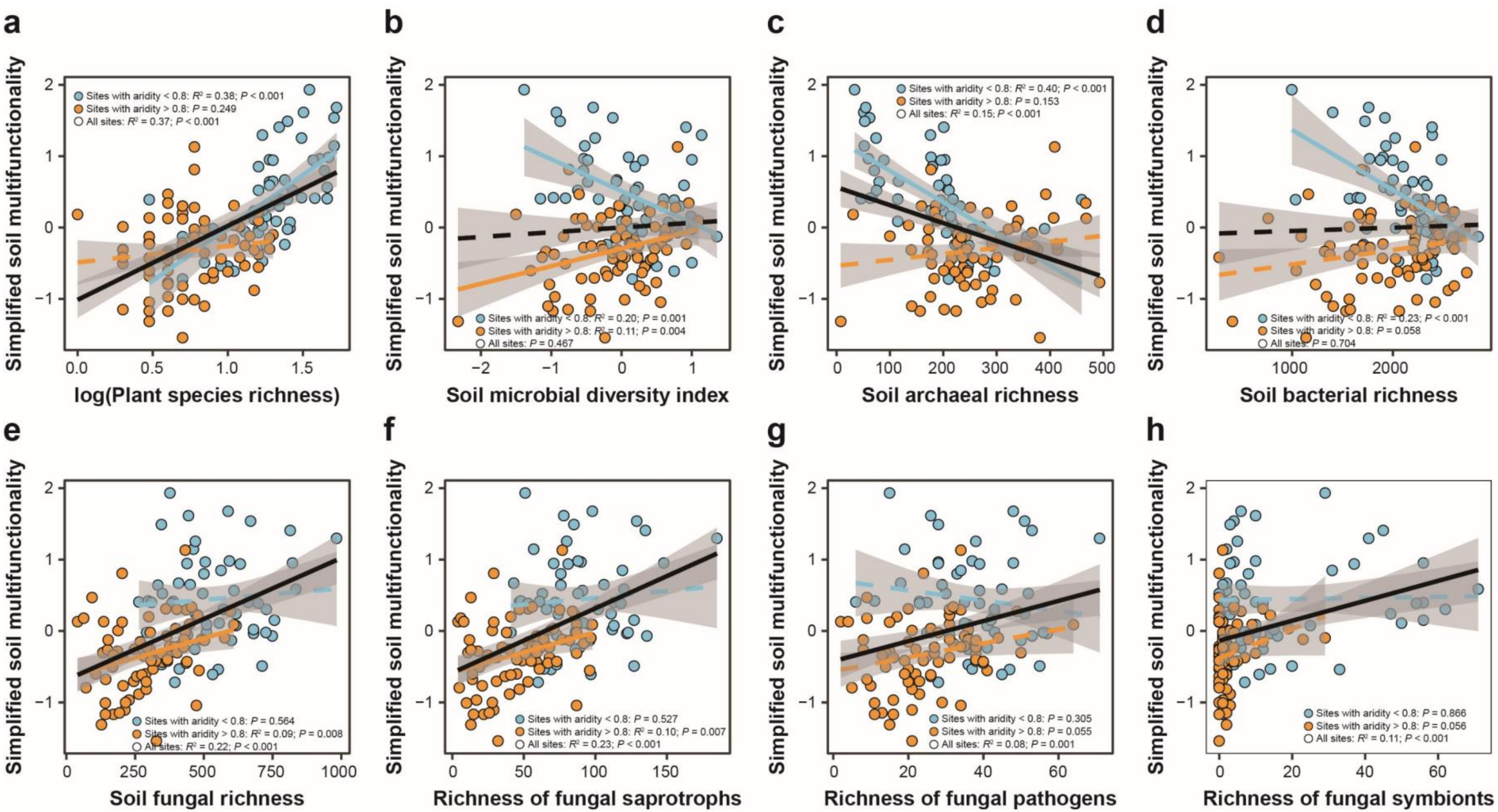

Supplementary Fig. 25. Relationships between plant or soil microbial diversity and simplified soil multifunctionality. a–h Relationships between log-transformed plant species richness (**a**), the soil microbial diversity index (**b**), soil archaeal richness (**c**), soil bacterial richness (**d**), soil fungal richness (**e**), richness of fungal saprotrophs (**f**), pathogens (**g**), and symbionts (**h**) and simplified soil multifunctionality at sites with aridity < 0.8 (N = 54) and > 0.8 (N = 76), as well as across all field sites (N = 130; the black lines). Lines represent the fitted linear OLS model. Solid and dashed lines denote statistically significant (two-sided $P \leq 0.05$) and non-significant (two-sided $P > 0.05$) relationships, respectively. Shaded areas denote the 95% confidence interval of the regression lines.

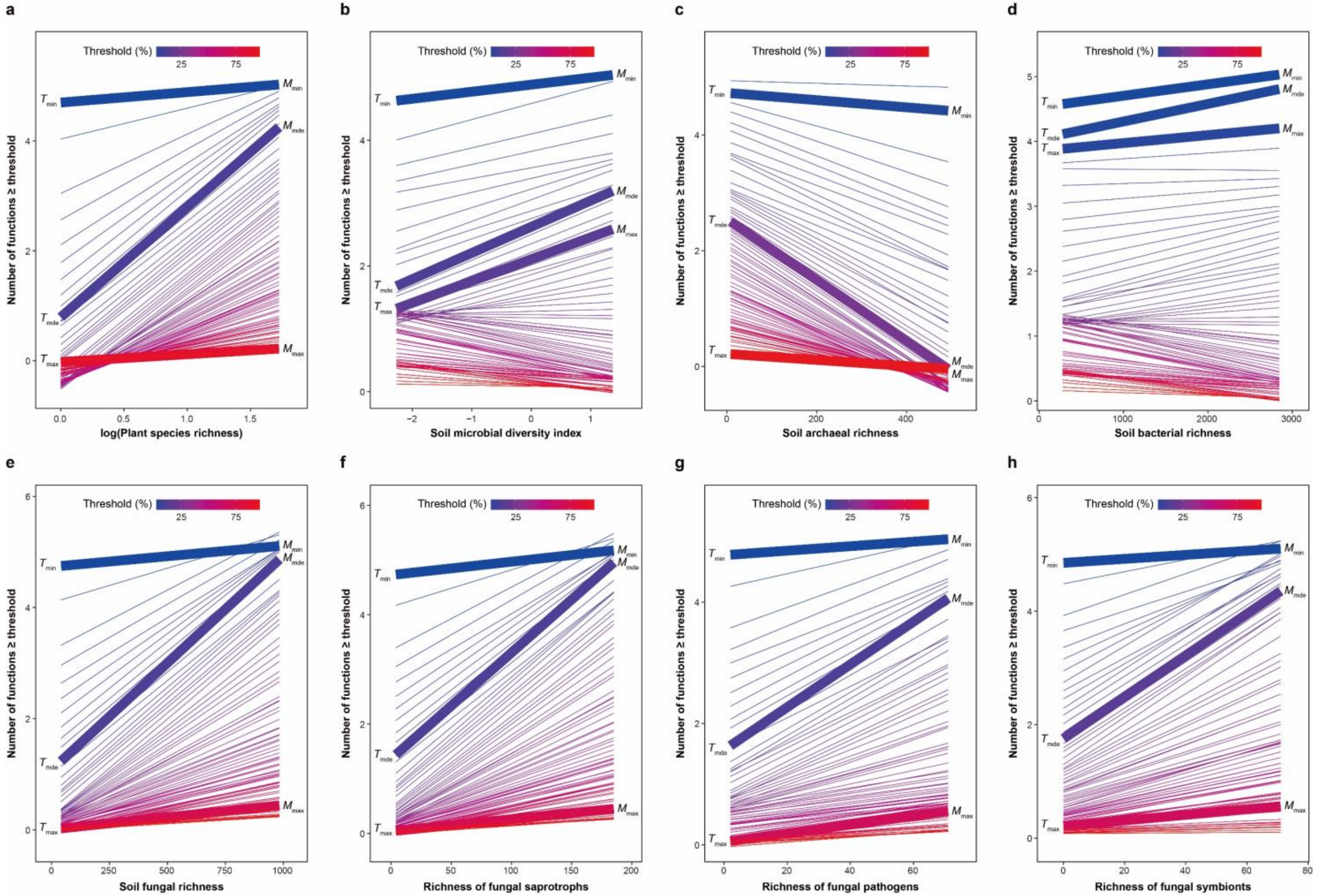

Supplementary Fig. 26. Relationships between biodiversity and simplified multiple-threshold multifunctionality. a–h Relationships between log-transformed plant species richness (**a**), the soil microbial diversity index (**b**), soil archaeal richness (**c**), soil bacterial richness (**d**), soil fungal richness (**e**), richness of fungal saprotrophs (**f**), pathogens (**g**), and symbionts (**h**) and the number of soil functions above multiple thresholds of the maximum observed soil function. Rest of legend as in Supplementary Fig. 4.

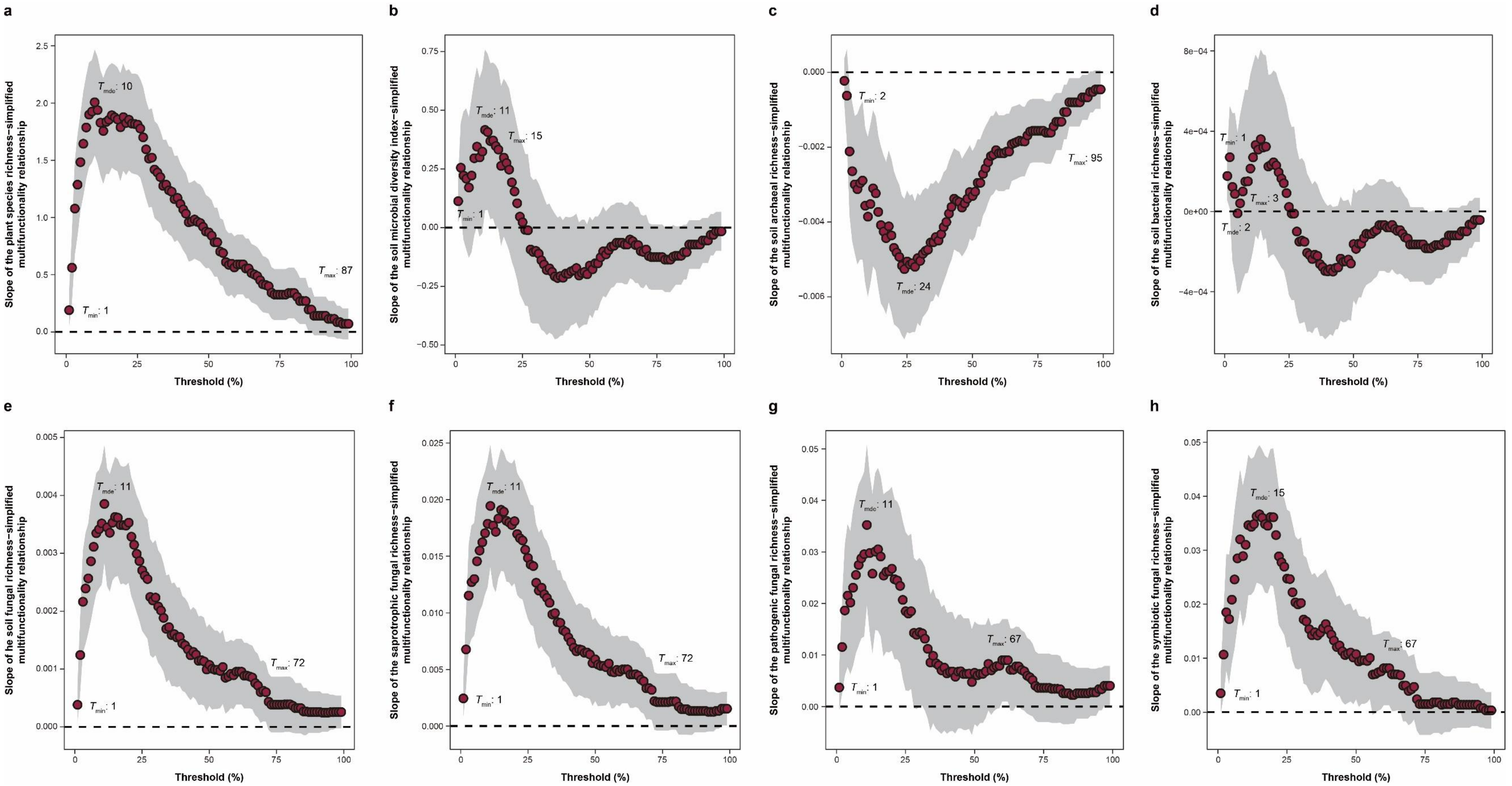

Supplementary Fig. 27. Slopes of the relationships between biodiversity and simplified multiple-threshold multifunctionality. a–h Slopes of the relationships between log-transformed plant species richness (**a**), the soil microbial diversity index (**b**), soil archaeal richness (**c**), soil bacterial richness (**d**), soil fungal richness (**e**), richness of fungal saprotrophs (**f**), pathogens (**g**), and symbionts (**h**) and the number of soil functions above the continuous thresholds from 1 to 99% of the maximum observed soil function. $T_{\min}$, T_{mde} and $T_{\max}$ correspond to those key thresholds depicted in Supplementary Fig. 26. Rest of legend as in Supplementary Fig. 4.

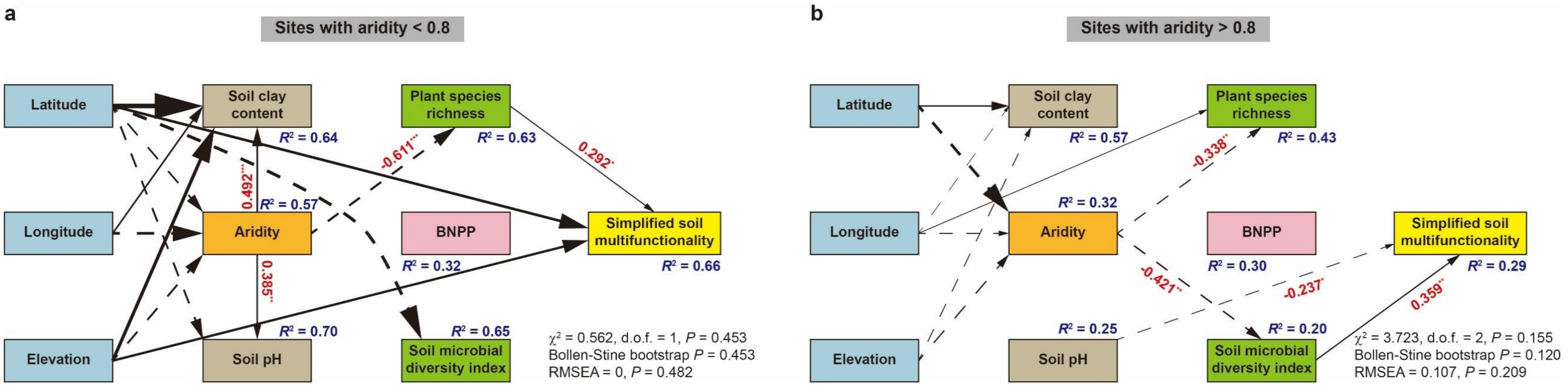

Supplementary Fig. 28. SEMs accounting for the hypothesized causal relationships between aridity, soil properties (pH and clay content), biodiversity (plant species richness and the soil microbial diversity index), BNPP and simplified soil multifunctionality. a,b SEMs are shown for sites with aridity < 0.8 (N = 54) and > 0.8 (N = 76). We only present significant relationships (two-sided $P < 0.05$) and their coefficients (numbers adjacent to arrows) for graphical simplicity. Latitude, longitude, and elevation of the field sites are included to account for the spatial structure of our dataset, and thus their coefficients are not included. An *a priori* model including all hypothesized causal relationships is available in Supplementary Fig. 17a, and all the rest of coefficients and their significance levels are available in Supplementary Table 8. For the SEM of sites with aridity > 0.8, we remove the relationship between soil pH and BNPP with a coefficient close to zero to improve its overall goodness of fit. Continuous and dashed arrows indicate positive and negative relationships, respectively. The thickness of the arrow is proportional to the magnitude of standardized path coefficients and indicative of the strength of the relationship. Asterisks indicate the significance level of each coefficient: *$P < 0.05$; **$P < 0.01$; ***$P < 0.001$. R^2 is the proportion of variance explained by the model. Goodness-of-fit statistics for each SEM are given (d.o.f., degrees of freedom; RMSEA, root mean squared error of approximation).

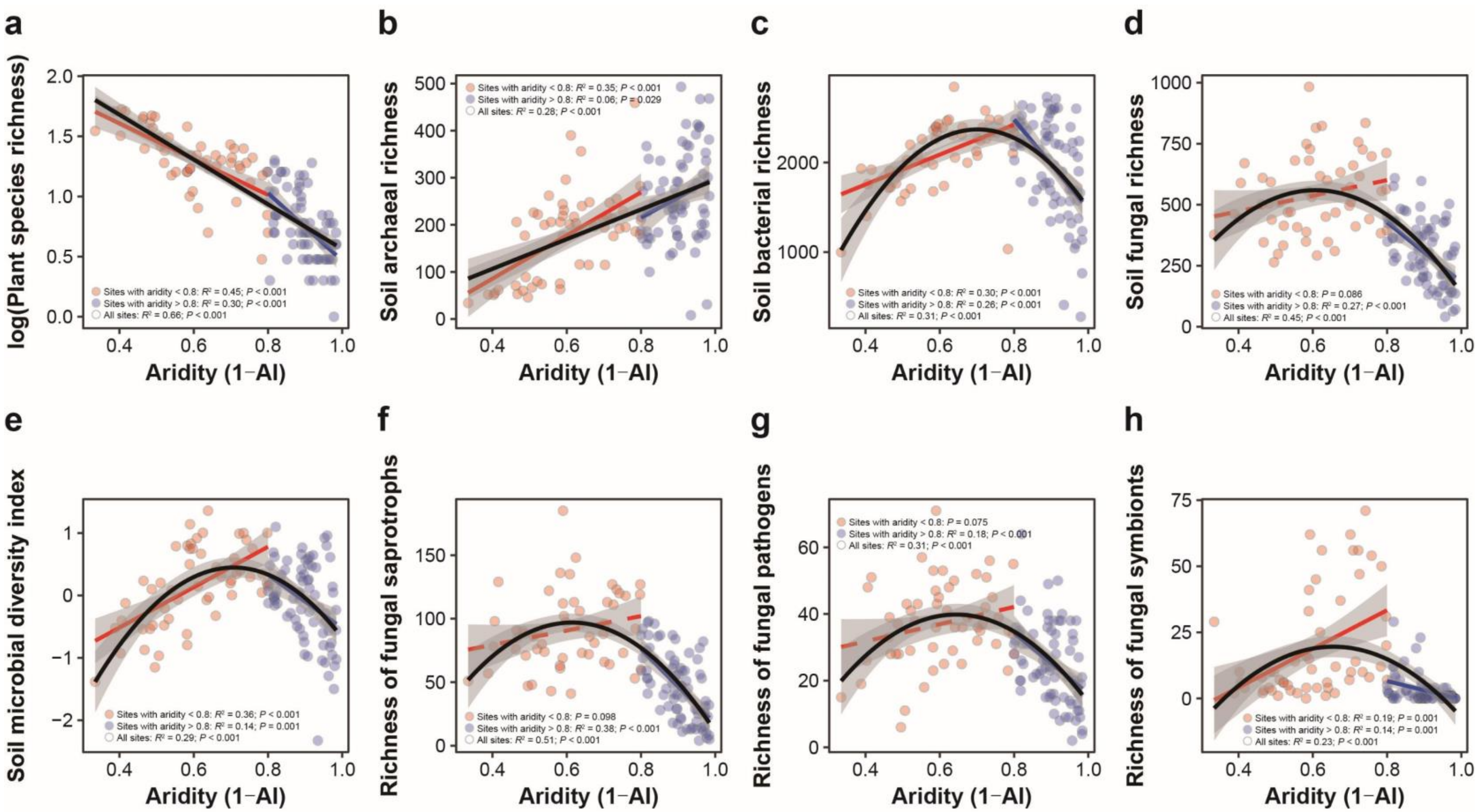

Supplementary Fig. 29. Relationships between aridity and each component of plant or soil microbial diversity. a–h Relationships between aridity and log-transformed plant species richness (**a**), soil archaeal richness (**b**), soil bacterial richness (**c**), soil fungal richness (**d**), the soil microbial diversity index (**e**), richness of fungal saprotrophs (**f**), pathogens (**g**), and symbionts (**h**) at sites with aridity < 0.8 ($N = 54$) and > 0.8 ($N = 76$), as well as across all field sites ($N = 130$; the black lines). Lines represent the fitted linear or quadratic OLS model. Model choice was based on AIC value. Differences in AIC (ΔAIC) values > 2 indicate that the models are different. Linear model was chosen when the ΔAIC values between linear and quadratic models were < 2. Solid and dashed lines denote statistically significant (two-sided $P \leq 0.05$) and non-significant (two-sided $P > 0.05$) relationships, respectively. Shaded areas denote the 95% confidence interval of the regression lines.

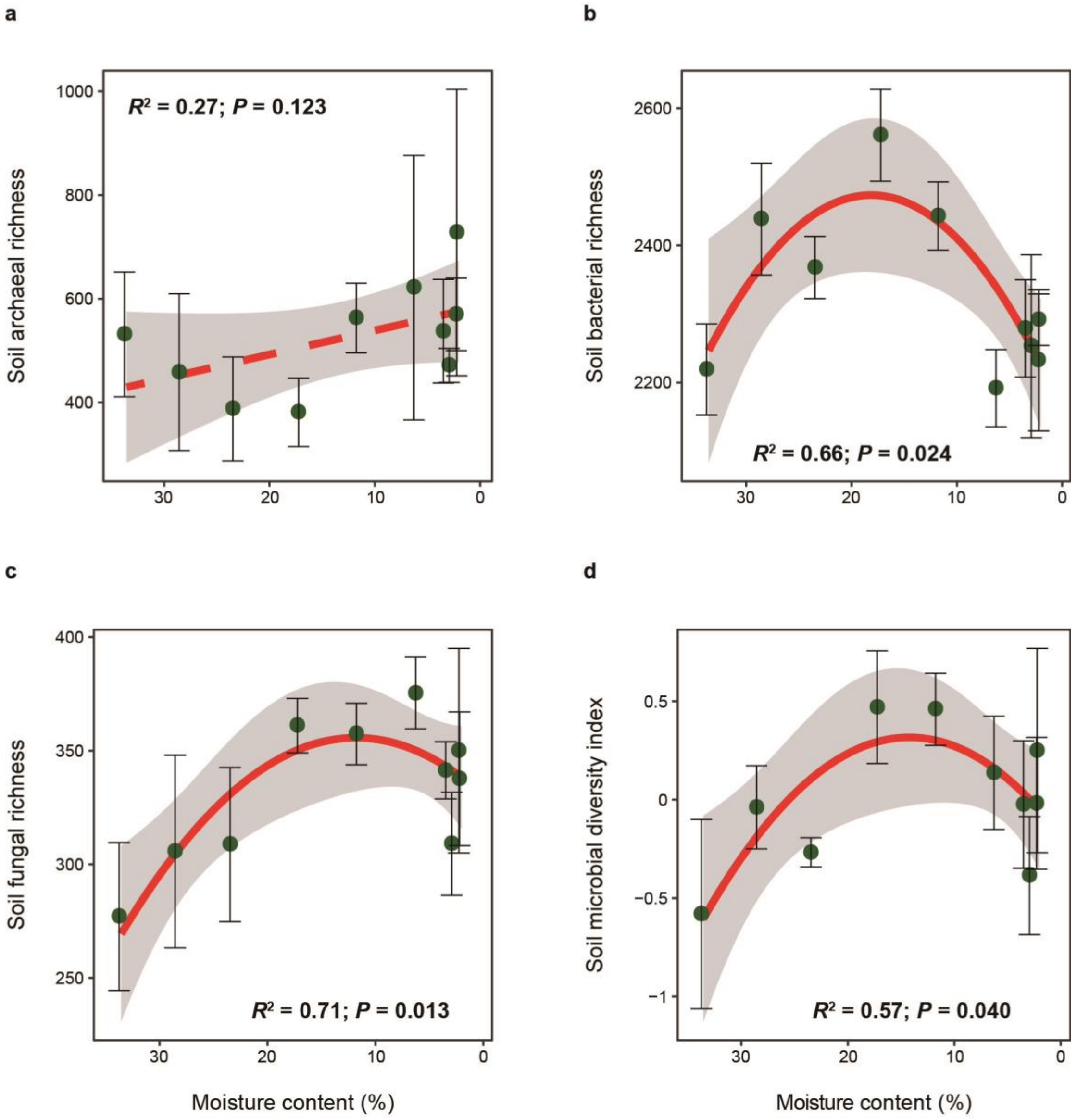

Supplementary Fig. 30. Relationships between moisture content and each component of soil microbial diversity for the experimental microcosms. a–d Relationships between moisture content and soil archaeal richness (**a**), soil bacterial richness (**b**), soil fungal richness (**c**), and the soil microbial diversity index (**d**). The red lines represent the fitted linear or quadratic OLS model. Dots represent means ± SE (N = 3 experimentally independent replicates). Solid and dashed lines denote statistically significant (two-sided $P \leq 0.05$) and non-significant (two-sided $P > 0.05$) relationships, respectively. Shaded areas denote the 95% confidence interval of the regression lines. Rest of legend as in Supplementary Fig. 29.

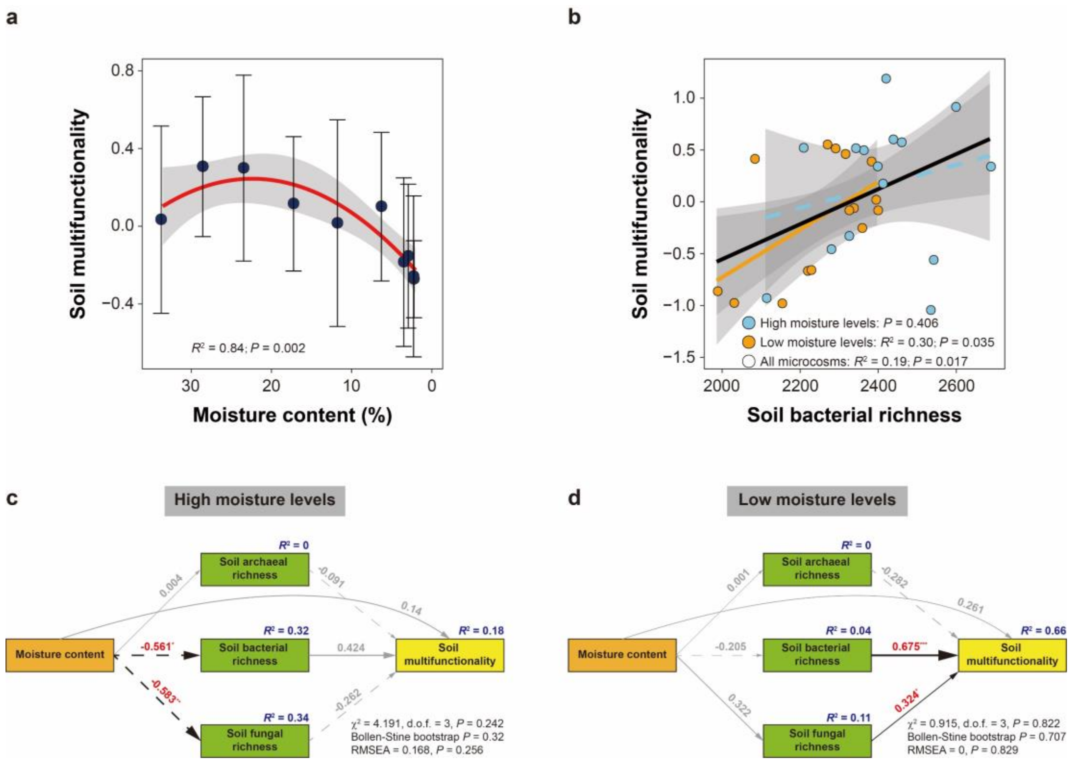

Supplementary Fig. 31. Relationships between moisture content, microbial diversity, and soil multifunctionality for the experimental microcosms. a Bivariate correlation between moisture content and soil multifunctionality. The red line represents the fitted quadratic OLS model. Model choice is based on ΔAIC value. Dots represent means ± SE (N = 3 experimentally independent replicates). The solid line denotes statistically significant (two-sided $P \leq 0.05$) relationship. The shaded area denotes the 95% confidence interval of the regression line. **b** Relationship between soil bacterial richness and multifunctionality at high (40–120% field capacity; N = 15) and low (3–20% field capacity; N = 15) moisture levels, as well as across all experimental microcosms (N = 30; the black line). Lines represent the fitted linear OLS model. Solid and dashed lines denote statistically significant (two-sided $P \leq 0.05$) and non-significant (two-sided $P > 0.05$) relationships, respectively. Shaded areas denote the 95% confidence interval of the regression lines. **c,d** SEMs accounting for the hypothesized direct and indirect relationships between moisture content, soil microbial diversity and multifunctionality at high (**c**; 40–120% field capacity; N = 15) and low (**d**; 3–20% field capacity; N = 15) moisture levels. An *a priori* model is available in Supplementary Fig. 17b. Black and gray arrows denote significant (two-sided $P < 0.05$) and non-significant (two-sided $P > 0.05$) relationships, respectively. Asterisks indicate the significant level of each coefficient: *$P < 0.05$; **$P < 0.01$; ***$P < 0.001$. Rest of legend as in Supplementary Fig. 28.

Supplementary Table 1. Description of the geographic, climatic, and soil characteristics of the field sites (*N* = 130).

Variable	Minimum	Maximum	Range	Median	Mean	SE.mean	Std.dev
Longitude (°E)	76.62	122.41	45.79	101.18	101.93	1.18	13.49
Latitude (°N)	35.89	50.70	14.81	41.96	42.28	0.33	3.74
Elevation (m a.s.l.)	204.00	3570.00	3366.00	1121.00	1294.00	68.00	781.00
MAT (ºC)	–4.30	12.80	17.10	5.70	5.05	0.34	3.86
MAP (mm $year^{-1}$)	21.00	453.00	432.00	167.00	195.46	11.49	130.99
MAE (mm $year^{-1}$)	688.00	1363.00	675.00	947.00	964.88	13.49	153.81
Aridity	0.33	0.98	0.65	0.82	0.78	0.015	0.17
Soil pH	5.82	9.65	3.84	8.47	8.32	0.066	0.75
Soil clay content (%)	7.00	27.00	20.00	18.00	17.86	0.35	4.01
SOC (g kg^{-1})	0.44	69.05	68.61	2.80	8.98	1.25	14.22
TN (g kg^{-1})	0.04	4.99	4.95	0.24	0.72	0.089	1.01
Ammonium (mg kg^{-1})	0	25.00	25.00	2.37	3.82	0.42	4.82
Nitrate (mg kg^{-1})	0.93	300.18	299.25	8.45	18.84	2.88	32.84
TP (g kg^{-1})	0.05	1.11	1.06	0.39	0.41	0.017	0.19
AP (mg kg^{-1})	0.35	22.67	22.32	2.69	3.37	0.24	2.78

MAT, mean annual temperature; MAP, mean annual precipitation; MAE, mean annual potential evapotranspiration; SOC, soil organic carbon; TN, total soil nitrogen; TP, total soil phosphorus; AP, soil available phosphorus.

Supplementary Table 2. Best models for each variable.

Variable	Linear AIC	Quadratic AIC	GAM AIC	AIC of threshold models			Best threshold model
				Stegmented	Step	Segmented	
DNA concentration	68.8	69.1	58.4	53.6	74.3	62.9	Stegmented
Soil organic carbon	128.4	123.7	117.0	116.9	143.6	118.2	Stegmented
Total soil nitrogen	111.9	113.7	106.6	104.0	131.9	109.8	Stegmented
Soil ammonium	76.0	77.7	73.4	70.2	68.2	76.3	Stegmented
Soil nitrate	166.8	159.9	161.3	155.8	161.1	157.6	Stegmented
Total soil phosphorus	5.2	–0.2	–24.5	–36.5	–2.4	–25.9	Stegmented
Soil available phosphorus	50.4	48.2	48.3	42.3	48.9	44.3	Stegmented
Soil multifunctionality	192.8	189.2	176.9	170.5	213.0	174.7	Stegmented
Standardized coefficients of relationship between PSR and soil multifunctionality	–19128.1	–19620.8	–22943.0	–22105.4	–21029.7	–21129.8	Stegmented
Standardized coefficients of relationship between SMDI and soil multifunctionality	6985.4	–4276.8	–13474.2	–14152.1	3637.9	–13816.2	Stegmented
Standardized coefficients of relationship between PSR interaction with aridity and soil multifunctionality	–12275.4	–20524.2	–26659.6	–	–	–	–
Standardized coefficients of relationship between SMDI interaction with aridity and soil multifunctionality	–20116.1	–25404.5	–29148.5	–	–	–	–
Simplified soil multifunctionality	188.6	186.8	183.6	179.5	212.0	180.1	Stegmented
Standardized coefficients of relationship between PSR and simplified soil multifunctionality	–24388.5	–25112.0	–28562.2	–27650.4	–27473.9	–28252.5	Segmented
Standardized coefficients of relationship between SMDI and simplified soil multifunctionality	4009.5	–5129.7	–14821.1	–15874.9	859.6	–14763.1	Stegmented

Standardized coefficients of relationship between PSR interaction with aridity and simplified soil multifunctionality	–12074.2	–14682.4	–29697.7	–	–	–	–
Standardized coefficients of relationship between SMDI interaction with aridity and simplified soil multifunctionality	–26502.8	–30835.1	–33218.9	–	–	–	–

Variables with their corresponding AIC values after fitting linear, nonlinear and threshold models are shown. Lower AIC values indicate a better fit of the model. PSR, plant species richness; SMDI, soil microbial diversity index; GAM, generalized additive model.

Supplementary Table 3. Values for indices generated by evaluating the relationships of soil multifunctionality and simplified soil multifunctionality with aridity and biodiversity using the multiple-threshold approach.

Variable	N_{func}	T_{min}	T_{max}	T_{mde}	R_{mde}	M_{min}	M_{max}	M_{mde}
Seven individual soil functions								
Aridity	7	1%	99%	10%	–6.46	6.75	0.01	2.71
Plant species richness	7	1%	99%	10%	2.68	7.07	0.28	6.07
Soil microbial diversity index	7	31%	87%	38%	–0.50	1.42	–0.03	0.77
Soil archaeal richness	7	2%	99%	23%	–0.01	6.18	–0.08	0.63
Soil bacterial richness	7	31%	86%	39%	–0.001	1.53	0.004	0.81
Soil fungal richness	7	1%	67%	11%	0.005	7.14	0.97	6.70
Richness of fungal saprotrophs	7	1%	72%	11%	0.02	7.22	0.74	6.81
Richness of fungal pathogens	7	1%	23%	11%	0.04	7.05	3.38	5.45
Richness of fungal symbionts	7	1%	32%	14%	0.05	7.12	2.83	6.47
Five individual soil functions excluding TP and TN								
Aridity	5	1%	97%	10%	–4.99	4.80	0.03	1.69
Plant species richness	5	1%	87%	10%	2.01	5.05	0.22	4.25
Soil microbial diversity index	5	1%	15%	11%	0.42	5.06	2.58	3.19
Soil archaeal richness	5	2%	95%	24%	–0.01	4.42	–0.04	0.01
Soil bacterial richness	5	1%	3%	2%	0.0003	5.07	4.20	4.81
Soil fungal richness	5	1%	72%	11%	0.004	5.13	0.44	4.87
Richness of fungal saprotrophs	5	1%	72%	11%	0.02	5.19	0.46	4.96
Richness of fungal pathogens	5	1%	67%	11%	0.04	5.04	0.56	4.06
Richness of fungal symbionts	5	1%	67%	15%	0.04	5.10	0.56	4.35

T_{min} and T_{max} represent the lowest and highest thresholds whose slopes are significantly different from zero, respectively. T_{mde} is the threshold with the steepest slope. All indices preceded by M indicate the number of soil functions achieving at the corresponding thresholds. R_{mde} denotes the slope calculated at T_{mde}. TP, total soil phosphorus; TN, total soil nitrogen.

Supplementary Table 4. Linear mixed-effects models for the relationships between multiple abiotic (aridity, soil pH and clay content) and biotic (BNPP, plant species richness, and soil archaeal, bacterial, and fungal richness) factors and soil multifunctionality at sites with aridity < 0.8 and > 0.8.

Term	df	ddf	MS	F	P	Estimate	VIF
Sites with aridity < 0.8 (N = 54); Random term is soil type; Conditional R^2 0.72; Marginal R^2 0.66							
Plant species richness	1	22.3	12.08	42.55	< 0.001	0.29	5.09
Soil fungal richness	1	35.7	0.07	0.23	0.633	0.33	2.42
Soil archaeal richness	1	31.9	2.69	9.48	0.004	–0.33	5.64
Soil bacterial richness	1	34.8	6.56	23.10	< 0.001	–0.04	6.05
Aridity	1	36.4	0.06	0.20	0.658	–0.17	4.62
BNPP	1	34.5	0.36	1.26	0.270	0.19	1.79
Soil pH	1	36.6	0.001	0.003	0.959	–0.16	3.95
Soil clay content	1	36.7	0.28	0.98	0.328	0.12	2.03
Longitude	1	29.9	1.35	4.74	0.037	–0.28	3.97
Plant species richness × Soil fungal richness	1	35.9	0.76	2.67	0.111	0.10	3.47
Plant species richness × Soil archaeal richness	1	36.9	0.49	1.71	0.199	–0.05	5.23
Plant species richness × Soil bacterial richness	1	36.4	0.23	0.82	0.371	0.18	8.63
Aridity × Plant species richness	1	35.9	0.22	0.77	0.387	0.38	4.61
Aridity × Soil fungal richness	1	36.2	0.23	0.81	0.374	–0.20	3.59
Aridity × Soil archaeal richness	1	36.2	2.49	8.78	0.005	0.54	6.61
Aridity × Soil bacterial richness	1	33.0	0.25	0.87	0.357	0.16	7.24
Sites with aridity > 0.8 (N = 76); Random term is soil type; Conditional R^2 0.32; Marginal R^2 0.31							
Soil fungal richness	1	56.8	5.15	6.28	0.015	0.12	2.73
Soil archaeal richness	1	57.0	0.69	0.84	0.363	0.22	2.08
Soil bacterial richness	1	56.9	0.14	0.18	0.677	0.04	3.61
Plant species richness	1	56.9	0.43	0.53	0.471	–0.20	2.62
Aridity	1	55.8	3.88	4.73	0.034	–0.30	2.54
BNPP	1	56.2	0.10	0.12	0.727	0.02	1.82
Soil pH	1	54.6	1.52	1.85	0.180	–0.21	1.60

Soil clay content	1	57.0	7.50	9.14	0.004	0.19	3.09
Elevation	1	56.9	0.01	0.01	0.933	0.16	4.26
Latitude	1	56.6	4.24	5.17	0.027	0.34	6.47
Longitude	1	56.9	0.14	0.17	0.678	–0.05	1.67
Plant species richness × Soil fungal richness	1	55.9	0.92	1.12	0.293	0.16	4.27
Plant species richness × Soil archaeal richness	1	54.9	0.14	0.16	0.687	0.04	2.71
Plant species richness × Soil bacterial richness	1	55.5	0.21	0.25	0.618	0.09	3.41
Aridity × Plant species richness	1	56.4	0.32	0.39	0.534	–0.05	2.14
Aridity × Soil fungal richness	1	57.0	0.91	1.11	0.297	0.12	3.45
Aridity × Soil archaeal richness	1	56.3	0.94	1.15	0.288	0.19	2.62
Aridity × Soil bacterial richness	1	56.5	0.16	0.20	0.660	0.12	5.17

Fixed terms are fitted sequentially (type-I sum of squares) as indicated in the table, and × denotes an interaction term. Soil and vegetation types are included as random terms. However, the term "vegetation type" is removed from the model in both cases because its variance is close to zero. To further address multicollinearity [the terms with VIF (variance inflation factor) values > 10 (ref. 1)], we removed the terms "Year", "Elevation", and "Latitude" from the model fitted for sites with aridity < 0.8 and the term "Year" from the model fitted for sites with aridity > 0.8. Marginal (variance explained by fixed terms) and conditional (variance explained by fixed and random terms) R^2 values are shown. Latitude, longitude, and elevation of the field sites are included to account for the spatial structure of our dataset. df, numerator degrees of freedom; ddf, denominator degrees of freedom; MS, mean squares; F, variance ratio; P, probability of type-I error (two-sided).

Supplementary Table 5. Standardized coefficients of all hypothesized causal relationships and their significance levels for SEMs described in Fig. 5 of the main text.

Structural equation models (SEMs)	Hypothesized response variables		Hypothesized predictors	Standardized coefficients	Two-sided *P*-value
Sites with aridity < 0.8 (N = 54)					
	Aridity	←	Latitude	–0.488	< 0.001
	Aridity	←	Longitude	–0.778	< 0.001
	Aridity	←	Elevation	–0.537	0.002
	Soil clay content	←	Latitude	1.245	< 0.001
	Soil clay content	←	Longitude	0.454	0.011
	Soil clay content	←	Elevation	0.910	< 0.001
	Soil clay content	←	Aridity	0.492	< 0.001
	Soil pH	←	Latitude	–0.535	0.007
	Soil pH	←	Longitude	–0.291	0.092
	Soil pH	←	Elevation	–0.319	0.101
	Soil pH	←	Aridity	0.385	0.003
	Soil pH	←	Soil clay content	0.028	0.825
	Plant species richness	←	Latitude	0.139	0.555
	Soil microbial diversity index	←	Latitude	–0.871	< 0.001
	Plant species richness	←	Longitude	0.101	0.612
	Soil microbial diversity index	←	Longitude	0.290	0.131
	Plant species richness	←	Elevation	–0.046	0.838
	Soil microbial diversity index	←	Elevation	–0.216	0.316
	Plant species richness	←	Aridity	–0.611	< 0.001
	Soil microbial diversity index	←	Aridity	0.193	0.203

	Plant species richness	←	Soil clay content	0.231	0.100
	Soil microbial diversity index	←	Soil clay content	0.186	0.170
	Plant species richness	←	Soil pH	0.130	0.398
	Soil microbial diversity index	←	Soil pH	0.237	0.112
	BNPP	←	Latitude	0.340	0.344
	BNPP	←	Longitude	–0.326	0.236
	BNPP	←	Elevation	–0.069	0.821
	BNPP	←	Aridity	–0.003	0.990
	BNPP	←	Soil clay content	0.237	0.231
	BNPP	←	Soil pH	–0.080	0.711
	BNPP	←	Plant species richness	0.229	0.220
	BNPP	←	Soil microbial diversity index	0.087	0.653
	Soil multifunctionality	←	Latitude	0.768	0.004
	Soil multifunctionality	←	Longitude	0.083	0.686
	Soil multifunctionality	←	Elevation	0.705	0.002
	Soil multifunctionality	←	Aridity	0.024	0.892
	Soil multifunctionality	←	Soil clay content	–0.078	0.597
	Soil multifunctionality	←	Soil pH	–0.252	0.113
	Soil multifunctionality	←	Plant species richness	0.357	0.011
	Soil multifunctionality	←	Soil microbial diversity index	0.170	0.232
	Soil multifunctionality	←	BNPP	0.029	0.777
Sites with aridity > 0.8 (N = 76)					
	Aridity	←	Latitude	–0.790	< 0.001
	Aridity	←	Longitude	–0.265	0.007

Aridity	←	Elevation	–0.366	0.019
Soil clay content	←	Latitude	0.446	0.002
Soil clay content	←	Longitude	–0.166	0.043
Soil clay content	←	Elevation	–0.257	0.045
Soil clay content	←	Aridity	–0.081	0.378
Soil pH	←	Latitude	0.245	0.222
Soil pH	←	Longitude	–0.103	0.355
Soil pH	←	Elevation	–0.269	0.122
Soil pH	←	Aridity	–0.223	0.067
Soil pH	←	Soil clay content	–0.187	0.221
Plant species richness	←	Latitude	0.278	0.117
Soil microbial diversity index	←	Latitude	–0.105	0.617
Plant species richness	←	Longitude	0.229	0.019
Soil microbial diversity index	←	Longitude	0.112	0.335
Plant species richness	←	Elevation	–0.079	0.608
Soil microbial diversity index	←	Elevation	–0.075	0.680
Plant species richness	←	Aridity	–0.338	0.002
Soil microbial diversity index	←	Aridity	–0.421	0.001
Plant species richness	←	Soil clay content	0.027	0.842
Soil microbial diversity index	←	Soil clay content	–0.168	0.292
Plant species richness	←	Soil pH	0.093	0.356
Soil microbial diversity index	←	Soil pH	0.045	0.707
BNPP	←	Latitude	0.236	0.235
BNPP	←	Longitude	0.131	0.241
BNPP	←	Elevation	0.125	0.458
BNPP	←	Aridity	–0.157	0.231

BNPP	←	Soil clay content	0.014	0.926
BNPP	←	Plant species richness	0.235	0.072
BNPP	←	Soil microbial diversity index	0.150	0.175
Soil multifunctionality	←	Latitude	0.415	0.038
Soil multifunctionality	←	Longitude	–0.089	0.430
Soil multifunctionality	←	Elevation	0.205	0.229
Soil multifunctionality	←	Aridity	–0.150	0.258
Soil multifunctionality	←	Soil clay content	0.224	0.132
Soil multifunctionality	←	Soil pH	–0.251	0.024
Soil multifunctionality	←	Plant species richness	–0.133	0.317
Soil multifunctionality	←	Soil microbial diversity index	0.291	0.009
Soil multifunctionality	←	BNPP	0.017	0.880

Supplementary Table 6. Linear mixed-effects model for the relationships between multiple biotic (BNPP, plant species richness and the soil microbial diversity index) and abiotic (aridity, soil pH and clay content) factors and simplified soil multifunctionality with considering soil and vegetation types as random terms.

**Term**	**df**	**ddf**	**MS**	***F***	***P***	**Estimate**	**VIF**
Random terms are soil and vegetation types; Conditional R^2 0.74; Marginal R^2 0.61							
Year	1	43.6	0.60	1.79	0.187	–0.13	2.06
Plant species richness	1	11.4	11.81	35.44	< 0.001	0.03	2.04
Soil microbial diversity index	1	104.8	0.13	0.40	0.527	0.32	2.45
Aridity	1	21.7	7.77	23.32	< 0.001	–0.27	1.92
BNPP	1	51.8	1.97	5.93	0.018	0.06	1.08
Soil pH	1	111.9	5.63	16.92	< 0.001	–0.29	1.62
Soil clay content	1	115.2	4.88	14.66	< 0.001	0.18	2.09
Elevation	1	111.6	0.03	0.10	0.758	0.13	2.28
Latitude	1	112.2	3.76	11.29	0.001	0.32	4.05
Longitude	1	113.4	0.06	0.19	0.668	0.03	1.67
Plant species richness × Soil microbial diversity index	1	112.3	0.26	0.77	0.382	0.28	4.45
Aridity × Plant species richness	1	106.8	1.18	3.54	0.063	–0.12	1.74
Aridity × Soil microbial diversity index	1	114.6	2.33	7.02	0.009	0.29	4.06

Fixed terms are fitted sequentially (type-I sum of squares) as indicated in Equation 2 in the main text, and × denotes an interaction term. Marginal (variance explained by fixed terms) and conditional (variance explained by fixed and random terms) R^2 values are shown. The term "Year" is first introduced into the model to eliminate the variation due to different sampling years. Latitude, longitude, and elevation of the field sites are included to account for the spatial structure of our dataset. df, numerator degrees of freedom; ddf, denominator degrees of freedom; MS, mean squares; *F*, variance ratio; *P*, probability of type-I error (two-sided).

Supplementary Table 7. Linear mixed-effects models for the relationships between multiple abiotic (aridity, soil pH and clay content) and biotic (BNPP, plant species richness, and soil archaeal, bacterial, and fungal richness) factors and simplified soil multifunctionality at sites with aridity < 0.8 and > 0.8.

Term	df	ddf	MS	*F*	*P*	Estimate	VIF
Sites with aridity < 0.8 (N = 54); Random term is soil type; Conditional R^2 0.77; Marginal R^2 0.71							
Plant species richness	1	24.0	12.00	52.30	< 0.001	0.24	4.91
Soil fungal richness	1	36.5	0.99	4.03	0.057	0.41	2.39
Soil archaeal richness	1	32.4	3.27	14.26	< 0.001	–0.26	5.49
Soil bacterial richness	1	34.7	6.14	26.76	< 0.001	–0.06	5.89
Aridity	1	36.7	0.44	1.90	0.176	–0.19	4.46
BNPP	1	34.4	0.50	2.19	0.148	0.20	1.79
Soil pH	1	36.4	0.22	0.98	0.330	–0.22	3.88
Soil clay content	1	36.4	0.35	1.54	0.223	0.14	2.01
Longitude	1	31.2	0.08	0.35	0.560	–0.12	3.92
Plant species richness × Soil fungal richness	1	36.1	0.83	3.62	0.065	0.07	3.43
Plant species richness × Soil archaeal richness	1	36.8	0.24	1.05	0.313	0.02	5.14
Plant species richness × Soil bacterial richness	1	36.5	0.48	2.07	0.158	0.17	8.49
Aridity × Plant species richness	1	35.9	0.17	0.74	0.396	0.37	4.49
Aridity × Soil fungal richness	1	35.9	0.09	0.39	0.534	–0.22	3.59
Aridity × Soil archaeal richness	1	36.3	2.48	10.81	0.002	0.56	6.43
Aridity × Soil bacterial richness	1	33.6	0.11	0.47	0.497	0.11	7.20
Sites with aridity > 0.8 (N = 76); Random terms are soil and vegetation types; Conditional R^2 0.57; Marginal R^2 0.25							
Year	1	55.5	5.31	6.86	0.011	–0.37	2.58
Soil fungal richness	1	53.7	5.53	7.15	0.010	0.07	2.97
Soil archaeal richness	1	54.6	0.61	0.78	0.380	0.20	2.52
Soil bacterial richness	1	55.0	0.30	0.39	0.537	0.10	3.45
Plant species richness	1	53.9	1.13	1.47	0.231	–0.07	2.78
Aridity	1	54.9	1.82	2.35	0.131	–0.21	2.48
BNPP	1	53.3	0.49	0.64	0.429	0.05	1.67

Soil pH	1	53.3	1.43	1.85	0.179	–0.21	1.65
Soil clay content	1	55.9	9.23	11.93	0.001	0.25	3.23
Elevation	1	54.8	0.40	0.52	0.475	–0.04	4.20
Latitude	1	55.2	1.61	2.08	0.155	0.24	6.25
Longitude	1	55.0	0.16	0.21	0.646	0.06	1.70
Plant species richness × Soil fungal richness	1	44.8	2.09	2.70	0.107	0.18	3.94
Plant species richness × Soil archaeal richness	1	54.6	0.02	0.02	0.883	0.12	2.80
Plant species richness × Soil bacterial richness	1	53.3	0.66	0.86	0.359	0.16	3.31
Aridity × Plant species richness	1	55.2	0.08	0.10	0.750	0.0003	2.03
Aridity × Soil fungal richness	1	55.2	0.32	0.41	0.524	0.07	3.33
Aridity × Soil archaeal richness	1	54.0	1.11	1.44	0.236	0.21	2.68
Aridity × Soil bacterial richness	1	54.4	0.08	0.10	0.749	0.08	5.03

Fixed terms are fitted sequentially (type-I sum of squares) as indicated in the table, and × denotes an interaction term. Soil and vegetation types are included as random terms. However, the term "vegetation type" is removed from the model fitted for sites with aridity < 0.8 because its variance is close to zero. To address multicollinearity, we removed the terms "Year", "Elevation", and "Latitude" from the model fitted for sites with aridity < 0.8. Marginal (variance explained by fixed terms) and conditional (variance explained by fixed and random terms) R^2 values are shown. The term "Year" is first introduced into the model fitted for sites with aridity > 0.8 to eliminate the variation due to different sampling years. Latitude, longitude, and elevation of the field sites are included to account for the spatial structure of our dataset. df, numerator degrees of freedom; ddf, denominator degrees of freedom; MS, mean squares; *F*, variance ratio; *P*, probability of type-I error (two-sided).

Supplementary Table 8. Standardized coefficients of all hypothesized causal relationships and their significance levels for SEMs described in Supplementary Fig. 28.

Structural equation models (SEMs)	Hypothesized response variables		Hypothesized predictors	Standardized coefficients	Two-sided *P*-value
Sites with aridity < 0.8 (N = 54)					
	Aridity	←	Latitude	–0.488	< 0.001
	Aridity	←	Longitude	–0.778	< 0.001
	Aridity	←	Elevation	–0.537	0.002
	Soil clay content	←	Latitude	1.245	< 0.001
	Soil clay content	←	Longitude	0.454	0.011
	Soil clay content	←	Elevation	0.910	< 0.001
	Soil clay content	←	Aridity	0.492	< 0.001
	Soil pH	←	Latitude	–0.535	0.007
	Soil pH	←	Longitude	–0.291	0.092
	Soil pH	←	Elevation	–0.319	0.101
	Soil pH	←	Aridity	0.385	0.003
	Soil pH	←	Soil clay content	0.028	0.825
	Plant species richness	←	Latitude	0.139	0.555
	Soil microbial diversity index	←	Latitude	–0.871	< 0.001
	Plant species richness	←	Longitude	0.101	0.612
	Soil microbial diversity index	←	Longitude	0.290	0.131
	Plant species richness	←	Elevation	–0.046	0.838
	Soil microbial diversity index	←	Elevation	–0.216	0.316
	Plant species richness	←	Aridity	–0.611	< 0.001
	Soil microbial diversity index	←	Aridity	0.193	0.203

Plant species richness	←	Soil clay content	0.231	0.100
Soil microbial diversity index	←	Soil clay content	0.186	0.170
Plant species richness	←	Soil pH	0.130	0.398
Soil microbial diversity index	←	Soil pH	0.237	0.112
BNPP	←	Latitude	0.340	0.344
BNPP	←	Longitude	–0.326	0.236
BNPP	←	Elevation	–0.069	0.821
BNPP	←	Aridity	–0.003	0.990
BNPP	←	Soil clay content	0.237	0.231
BNPP	←	Soil pH	–0.080	0.711
BNPP	←	Plant species richness	0.229	0.220
BNPP	←	Soil microbial diversity index	0.087	0.653
Simplified soil multifunctionality	←	Latitude	0.749	0.004
Simplified soil multifunctionality	←	Longitude	0.237	0.232
Simplified soil multifunctionality	←	Elevation	0.661	0.002
Simplified soil multifunctionality	←	Aridity	0.004	0.981
Simplified soil multifunctionality	←	Soil clay content	–0.043	0.765
Simplified soil multifunctionality	←	Soil pH	–0.292	0.056
Simplified soil multifunctionality	←	Plant species richness	0.292	0.030
Simplified soil multifunctionality	←	Soil microbial diversity index	0.194	0.142
Simplified soil multifunctionality	←	BNPP	0.046	0.637
Sites with aridity > 0.8 (N = 76)				
Aridity	←	Latitude	–0.790	< 0.001
Aridity	←	Longitude	–0.265	0.007

Aridity	←	Elevation	–0.366	0.019
Soil clay content	←	Latitude	0.446	0.002
Soil clay content	←	Longitude	–0.166	0.043
Soil clay content	←	Elevation	–0.257	0.045
Soil clay content	←	Aridity	–0.081	0.378
Soil pH	←	Latitude	0.245	0.222
Soil pH	←	Longitude	–0.103	0.355
Soil pH	←	Elevation	–0.269	0.122
Soil pH	←	Aridity	–0.223	0.067
Soil pH	←	Soil clay content	–0.187	0.221
Plant species richness	←	Latitude	0.278	0.117
Soil microbial diversity index	←	Latitude	–0.105	0.617
Plant species richness	←	Longitude	0.229	0.019
Soil microbial diversity index	←	Longitude	0.112	0.335
Plant species richness	←	Elevation	–0.079	0.608
Soil microbial diversity index	←	Elevation	–0.075	0.680
Plant species richness	←	Aridity	–0.338	0.002
Soil microbial diversity index	←	Aridity	–0.421	0.001
Plant species richness	←	Soil clay content	0.027	0.842
Soil microbial diversity index	←	Soil clay content	–0.168	0.292
Plant species richness	←	Soil pH	0.093	0.356
Soil microbial diversity index	←	Soil pH	0.045	0.707
BNPP	←	Latitude	0.236	0.235
BNPP	←	Longitude	0.131	0.241
BNPP	←	Elevation	0.125	0.458
BNPP	←	Aridity	–0.157	0.231

BNPP	←	Soil clay content	0.014	0.926
BNPP	←	Plant species richness	0.235	0.072
BNPP	←	Soil microbial diversity index	0.150	0.175
Simplified soil multifunctionality	←	Latitude	0.308	0.129
Simplified soil multifunctionality	←	Longitude	0.085	0.458
Simplified soil multifunctionality	←	Elevation	–0.022	0.898
Simplified soil multifunctionality	←	Aridity	–0.081	0.547
Simplified soil multifunctionality	←	Soil clay content	0.166	0.272
Simplified soil multifunctionality	←	Soil pH	–0.237	0.035
Simplified soil multifunctionality	←	Plant species richness	–0.216	0.109
Simplified soil multifunctionality	←	Soil microbial diversity index	0.359	0.001
Simplified soil multifunctionality	←	BNPP	0.071	0.543

Supplementary Table 9. Allometric models for estimating the aboveground and root biomass of dominant shrub species investigated in the field study.

Dominant shrub species	Model	*N*	R^2	*P*	Reference
Nitraria tangutorum	$B_{AGB} = 0.021 \times (C \times H)^{0.870}$	20	0.83	< 0.001	2
	$B_{BGB} = 0.87e\text{-}02 \times (C \times H)^{0.870}$	20	0.83	< 0.001	2
Suaeda dendroides	$B_{AGB} = 0.188 \times (C \times H)^{0.713}$	15	0.94	< 0.001	2
	$B_{BGB} = 0.136 \times (C \times H)^{0.713}$	15	0.94	< 0.001	2
Anabasis aphylla	$B_{AGB} = 0.084 \times (C \times H)^{0.785}$	20	0.97	< 0.001	2
	$B_{BGB} = 0.085 \times (C \times H)^{0.785}$	20	0.97	< 0.001	2
Calligonum rubicundum	$B_{AGB} = 0.727e\text{-}04 \times (C \times H)^{1.165}$	12	0.99	< 0.001	2
	$B_{BGB} = 0.709e\text{-}04 \times (C \times H)^{1.165}$	21	0.99	< 0.001	2
Haloxylon ammodendron	$B_{AGB} = 0.3628 \times (C \times H)^{0.9605}$	20	0.96	< 0.001	3
	$B_{BGB} = 0.8737 \times B_{AGB}^{0.9394}$	20	0.90	< 0.001	3
Kalidium foliatum	$B_{AGB} = -5.445 + 0.971 \times \ln(C \times H)$	34	0.92	< 0.001	4
	$B_{BGB} = -3.990 + 0.894 \times \ln(C)$	34	0.83	< 0.001	4
Sarcozygium xanthoxylon	$B_{AGB} = -2.091 + 0.686 \times \ln(C \times H)$	33	0.81	< 0.001	4
	$B_{BGB} = -2.163 + 0.687 \times \ln(C \times H)$	33	0.75	< 0.001	4
Artemisia ordosica	$B_{AGB} = -7.619 + 1.054 \times \ln(C \times H)$	34	0.95	< 0.001	4
	$B_{BGB} = -4.417 + 1.153 \times \ln(C \times H)$	37	0.92	< 0.001	4
Reaumuria songonica	$B_{AGB} = -3.895 + 1.027 \times \ln(C)$	34	0.90	< 0.001	4
	$B_{BGB} = -3.665 + 1.018 \times \ln(C)$	34	0.83	< 0.001	4
Salsola passerina	$\ln(B_{AGB}) = -8.025 + 1.172 \times \ln(C \times H)$	189	0.86	< 0.001	4
	$\ln(B_{BGB}) = -11.292 + 1.362 \times \ln(C \times H)$	84	0.83	< 0.001	4

These models are developed in previous studies that were conducted in the same regions as investigated in this study, and the references are given. B_{AGB},

aboveground biomass; B_{BGB}, root biomass; *C*, canopy cover of each selected individual; *H*, height of each selected individual; *N*, the total number of individuals used to fit the model; R^2, coefficient of determination of the model; *P*, significance level of the model (two-sided).

Supplementary Table 10. A complete list of all primers used in this study.

Microbial taxa	Primer name	Sequences
Archaea	Arch344F/Arch915R	5'-ACGGGGYGCAGCAGGCGCGA-3'/5'-GTGCTCCCCCGCCAATTCCT-3'
Bacteria	338F/806R	5'-ACTCCTACGGGAGGCAGCAG-3'/5'-GGACTACHVGGGTWTCTAAT-3'
Fungi	ITS1F/ITS2	5'-CTTGGTCATTTAGAGGAAGTAA-3'/5'-GCTGCGTTCTTCATCGATGC-3'

Supplementary Table 11. Test for the distribution of variables involving detection of aridity thresholds.

Variable	AIC from fitting two modes	AIC from fitting one mode	ΔAIC
DNA concentration	340.9	353.1	–12.2
Soil organic carbon	19.9	25.8	–5.9
Total soil nitrogen	202.3	212.5	–10.2
Soil ammonium	–320.7	–293.2	–27.5
Soil nitrate	523.1	535.0	–11.9
Total soil phosphorus	400.6	412.9	–12.3
Soil available phosphorus	464.3	476.9	–12.6
Soil multifunctionality	–375.0	–105.7	–269.3
Standardized coefficients of relationship between PSR and soil multifunctionality	–6978.5	–6734.0	–244.5
Standardized coefficients of relationship between SMDI and soil multifunctionality	9499.0	9970.3	–471.3
Simplified soil multifunctionality	266.6	267.6	–1.0
Standardized coefficients of relationship between PSR and simplified soil multifunctionality	–16214.7	–15998.3	–216.4
Standardized coefficients of relationship between SMDI and simplified soil multifunctionality	6491.3	7067.2	–575.9

ΔAIC, differences in AIC values from fitting two modes (bimodal) vs. fitting one mode (unimodal). Negative values of ΔAIC indicate that the variables present unimodal distributions[5]. PSR, plant species richness; SMDI, soil microbial diversity index.

Supplementary References

1. Hair, J. F., Black, W. C., Babin, B. J., Anderson, R. E. & Tatham, R. L. *Multivariate Data Analysis* (Prentice Hall, New Jersey, 1998).

2. Tao, Y. & Zhang, Y. M. Evaluation of vegetation biomass carbon storage in deserts of Central Asia. *Arid Land Geogr.* **36,** 615–622 (2013).

3. Tao, Y. & Zhang, Y. M. Multi-scale biomass estimation of desert shrubs: a case study of *Haloxylon ammodendron* in the Gurbantunggut Desert, China. *Acta Pratac. Sin.* **22,** 1–10 (2013).

4. Yang, H. T., Wang, Z. R., Tan, H. J. & Gao, Y. H. Allometric models for estimating shrub biomass in desert grassland in northern China. *Arid Land Res. Manag.* **31,** 283–300 (2017).

5. Berdugo, M. et al. Global ecosystem thresholds driven by aridity. *Science* **367,** 787–790 (2020).
